# Multi-omics approaches for understanding gene-environment interactions in noncommunicable diseases: techniques, translation, and equity issues

**DOI:** 10.1186/s40246-025-00718-9

**Published:** 2025-01-31

**Authors:** Robel Alemu, Nigussie T. Sharew, Yodit Y. Arsano, Muktar Ahmed, Fasil Tekola-Ayele, Tesfaye B. Mersha, Azmeraw T. Amare

**Affiliations:** 1 Program in Medical and Population Genetics, Broad Institute of MIT and Harvard, Cambridge, MA USA; 2 Anderson School of Management, University of California Los Angeles, Los Angeles, CA USA; 3 Adelaide Medical School, Faculty of Health and Medical Sciences, The University of Adelaide, Adelaide, Australia; 4 Alpert Medical School, Lifespan Health Systems, Brown University, WarrenProvidence, Rhode Island, USA; 5 Epidemiology Branch, Division of Population Health Research, Division of Intramural Research, Eunice Kennedy Shriver National Institute of Child Health and Human Development, National Institutes of Health, Bethesda, MD USA; 6 Department of Pediatrics, Cincinnati Children’s Hospital Medical Center, University of Cincinnati College of Medicine, Cincinnati, OH USA

## Abstract

Non-communicable diseases (NCDs) such as cardiovascular diseases, chronic respiratory diseases, cancers, diabetes, and mental health disorders pose a significant global health challenge, accounting for the majority of fatalities and disability-adjusted life years worldwide. These diseases arise from the complex interactions between genetic, behavioral, and environmental factors, necessitating a thorough understanding of these dynamics to identify effective diagnostic strategies and interventions. Although recent advances in multi-omics technologies have greatly enhanced our ability to explore these interactions, several challenges remain. These challenges include the inherent complexity and heterogeneity of multi-omic datasets, limitations in analytical approaches, and severe underrepresentation of non-European genetic ancestries in most omics datasets, which restricts the generalizability of findings and exacerbates health disparities. This scoping review evaluates the global landscape of multi-omics data related to NCDs from 2000 to 2024, focusing on recent advancements in multi-omics data integration, translational applications, and equity considerations. We highlight the need for standardized protocols, harmonized data-sharing policies, and advanced approaches such as artificial intelligence/machine learning to integrate multi-omics data and study gene-environment interactions. We also explore challenges and opportunities in translating insights from gene-environment (GxE) research into precision medicine strategies. We underscore the potential of global multi-omics research in advancing our understanding of NCDs and enhancing patient outcomes across diverse and underserved populations, emphasizing the need for equity and fairness-centered research and strategic investments to build local capacities in underrepresented populations and regions.

**Supplementary Information:**

The online version contains supplementary material available at 10.1186/s40246-025-00718-9.

## Introduction

Non-communicable diseases (NCDs), such as cardiovascular diseases, cancers, chronic respiratory diseases, diabetes, mental health disorders, and other complex diseases, pose a significant and growing global health challenge [1]. Annually, NCDs account for 41 million deaths, constituting 60% of Disability Adjusted Life Years (DALYs), 81% of Years Lived with Disability (YLDs), and 74% of all global fatalities [2, 21], making them the primary cause of disease burden and death worldwide [1]. For example, cardiovascular diseases alone claim 17.9 million lives each year, followed by cancers (9.3 million), chronic respiratory diseases (4.1 million), and diabetes-related conditions (2.0 million), together accounting for over 80% of all premature NCD deaths [2]. Economically, the cumulative global burden of NCDs from 2010 to 2030 is estimated to exceed USD 47 trillion—a figure that represents 75% of the global GDP in 2010 [3]. This rise in NCDs can largely be attributed to rapid unplanned urbanization, the globalization of unhealthy lifestyles, and an aging population [2, 4].

NCDs affect individuals across all demographics and countries, with a disproportionately severe impact on low- and middle-income countries, where over three-quarters of global NCD-related deaths, approximately 31.4 million, occur annually [5]. These diseases arise from complex interactions between genetic and environmental— including physical inactivity, unhealthy diets, obesity, and the use of tobacco or alcohol [6, 7]. Although NCDs typically manifest in adulthood, their roots are often traced back to behaviors and conditions established during childhood and adolescence [8, 9]. The burden of NCDs alongside existing infectious diseases poses significant economic stability and development challenges, exacerbating poverty and straining health systems, reducing resilience to emergencies such as infectious disease outbreaks and natural disasters [1, 3]. Furthermore, the high burden of NCDs is a major obstacle to progress towards the 2030 Agenda for Sustainable Development, specifically the target to reduce premature mortality from the four principal NCDs (cancers, cardiovascular diseases, chronic respiratory diseases, and diabetes) by one-third by 2030 [2, 3, 10].

Family- and population-based studies have revealed that most NCDs possess substantial genetic components, with diseases such as coronary artery disease (CAD) [11] and autism spectrum disorder (ASD) [12] demonstrating high heritability, estimated at approximately 50% and 80%, respectively. Most NCDs are predominantly polygenic, involving numerous genetic variants that each contribute subtly to overall disease risk [13–[15]. Advances in omics technologies, particularly genome-wide association studies (GWAS), have successfully identified many genetic variants linked to NCDs [15–[18]. However, our understanding of the genetic etiology of NCDs remains incomplete [8, 9, 96]. There are several challenges, including the 'missing heritability problem,' where known genetic variants associated with a disease/trait account for only a small fraction of the expected heritability [19–[23]. Additionally, pinpointing the true causal variants that contribute to disease mechanisms has proven difficult due to the complex linkage disequilibrium (LD) structure of GWAS nominated variants, which limits thier clinical utility [24–[26]. Recent advances in whole genome sequencing (WGS) studies have begun to elucidate the role of rare genetic variants in NCDs, while also offering additional insights into the contribution of common variants through improved resolution and comprehensive genomic coverage. Despite these insights, rare variants do not fully explain the missing heritability in NCDs, underscoring their complexity and multi-factorial nature [27]. A key factor that may explain this missing heritability is the complex interplay between genetic variants and environmental factors—often called gene-environment (GxE) interactions [21, 21–[30]. In this context, an ‘environment’ could be any endogenous or exogenous non-genetic factor that influences the risk of developing NCDs [31].

To fully understand the complex GxE interactions that underpin the biological basis of NCDs, it is essential to integrate information across multiple levels [32–[36]. This integration encompasses molecular profiles from the genome, epigenome, transcriptome, proteome, metabolome, lipidome, and microbiome—collectively referred to as multi-omics—along with environmental exposures known as the exposome. Rapid advancements in computational methodology have made the integration of multi-omics including high-throughput sequencing, mass spectrometry, smart wearable devices, and expanded electronic health records (EHRs) data increasingly feasible [33, 37, 38]. Such omics integration generates comprehensive data at an unprecedented speed and scale, enhancing our understanding of disease mechanisms and revolutionizing precision medicine [37, 39, 40] by enabling targeted prevention, precise diagnostics, personalized treatments, and accurate prognosis [37].

The multi-omics approach requires innovative integration methods that combine information from diverse omics data sources [35, 41, 42]. These methods facilitate the assessment of information flow from one omics layer to another and help elucidate the intricate interplay between various molecular profiles. Recently, advances in statistical modeling and machine learning have enabled more effective integration of omics data, which is crucial for tackling the complexity of NCDs [37, 38, 43, 44]. Despite this potential, several significant challenges remain. A primary obstacle is deciding which omics layer to prioritize in multi-omics studies [37]. While many researchers adopt a genome-first approach, the optimal strategy may vary depending on the specific disease and available data. Another challenge is the lack of genetic diversity in most multi-omics datasets, as most of these datasets have predominantly been based on samples of European genetic ancestry [45–[48]. Studies have shown that results derived from predominantly European datasets often do not translate well to individuals of non-European ancestry, potentially exacerbating health disparities by limiting research benefits to certain groups [48, 49]. Furthermore, the heterogeneity and massive scale of multi-omics datasets pose substantial challenges in data integration, requiring significant computational resources, skills, and advanced analytical techniques [50].

In this scoping review, we examine the multi-omics literature comprehensively, specifically focusing on NCDs and omics (multi-omics) diversity. Our primary goal is to assess the current landscape of global multi-omics data as it relates to NCDs, summarizing key advances in data integration techniques that enable a deeper understanding of the intricate GxE interactions at play. We will delve into the significant role of multi-omics research in elucidating the complex pathways influencing the development, progression, and response to treatment in NCDs. Next, we illustrate practical translational applications and point out critical limitations currently facing the field. Additionally, we discuss the transformative potential of global multi-omics research initiatives in advancing precision medicine, specifically in tailoring prevention, diagnosis, and treatment strategies to individual genetic and environmental profiles. Lastly, we propose directions to address existing challenges of multi-omics research to enhance our understanding of the biological mechanisms of NCDs and development of effective interventions.

## Omics technologies for unraveling GxE interactions in NCDs

Research has consistently demonstrated that the risk of developing most NCDs and the effectiveness of treatments are influenced by both the independent effects of an individual's genetic makeup and various environmental exposures, as well as by the potential synergistic or antagonistic interactions between these two factors [13, 14, 36, 51]. One type of GxE interaction occurs when an individual’s genotype modulates the effect of environmental exposure on disease risk. For example, certain genetic variants may alter the risk of developing Parkinson's disease in individuals exposed to organophosphate pesticides [52, 53]. Conversely, another form of GxE interaction happens when the influence of a genotype on disease risk changes with different environmental exposures [28]. A notable case is how the impact of the *FTO* gene on body mass index (BMI) can significantly vary depending on lifestyle factors such as physical activity, diet, alcohol consumption, and sleep duration [54]. These examples underscore the dynamic interplay between our largely static genetic code and the responsive molecular layers of the genome and epigenome. These layers dynamically respond to environmental changes, affecting gene expression and cellular functions, representing key mechanisms through which GxE interactions manifest.

Omics technologies— powered by advances in high-throughput sequencing technologies such as next-generation sequencing (NGS) and rapidly expanding electronic data (exosomes), enable a comprehensive analysis of various biological systems [55]. Each technology focuses on a different aspect: genomics and epigenomics explore genetic and epigenetic variations; transcriptomics examines gene expression dynamics; proteomics investigates protein functions and interactions; metabolomics assesses metabolic responses; exposomics evaluates lifelong environmental exposures. While each technology excels at quantifying specific types of biomolecules, the complete picture of disease mechanisms often involves intricate molecular machinery such as transcriptional and translational regulation, RNA and peptide degradation, posttranslational modifications, and molecular transport [55, 56]. Thus, focusing solely on one type of omics data can overlook critical interactions between these processes.

### Genomics

**Fig. 1:**
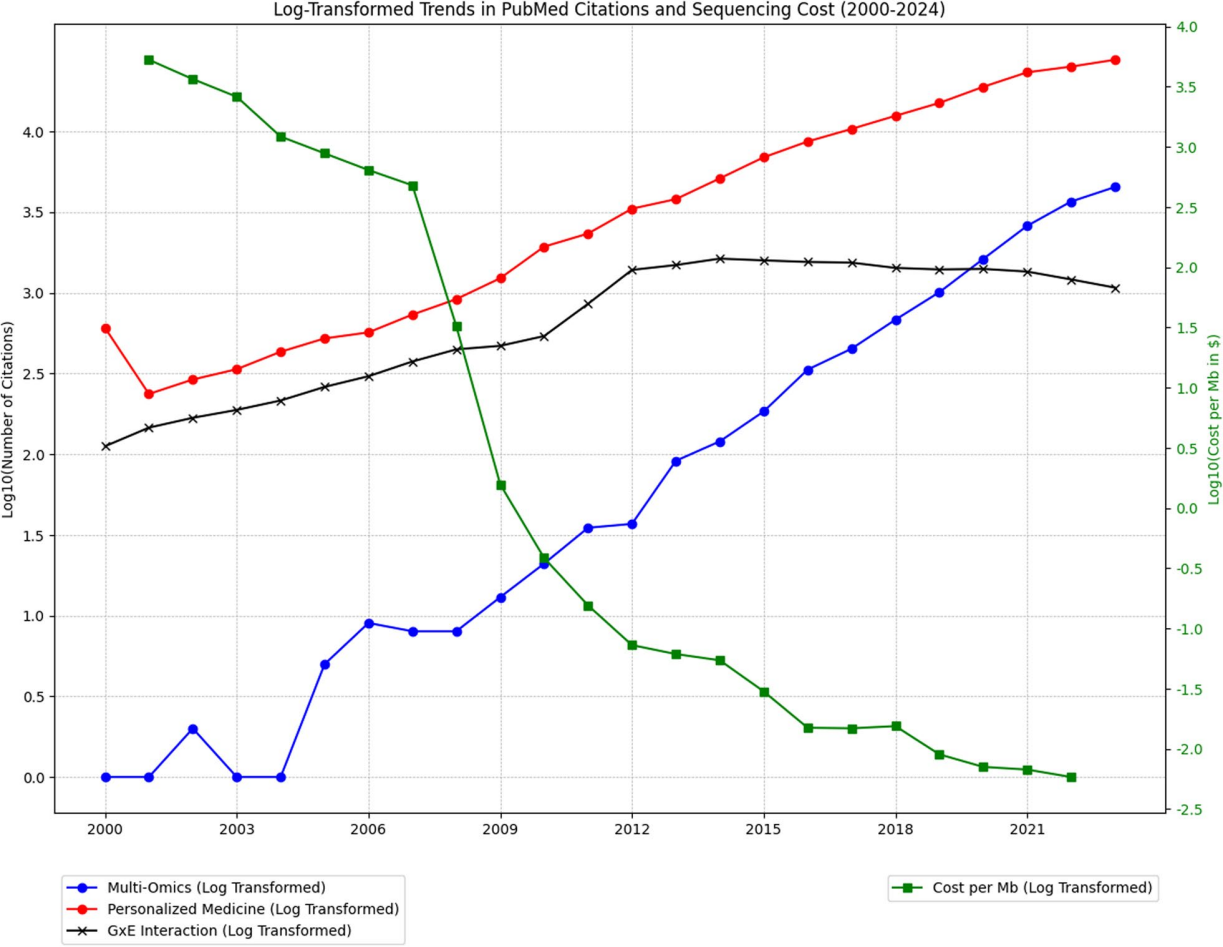
Log-Transformed Trends in PubMed Citation Frequencies and Sequencing Costs (2000–2024). This figure illustrates trends in sequencing costs and PubMed citation frequencies for key terms (“multi-omics,” “personalized/precision medicine,” and “gene-environment (GxE) interactions”) from 2000 to 2024. Citation data were derived using a Python-based web scraping approach that sends HTTP requests to PubMed and parses the HTML response using the BeautifulSoup library. For each year, search queries targeted keywords in the title/abstract, filtering results by publication year to extract the annual citation count. Sequencing cost data were sourced from the National Human Genome Research Institute’s (NHGRI) Genome Sequencing Program (GSP) database (Wetterstrand KA, www.genome.gov/sequencingcostsdata; accessed June 17, 2024). Both the citation frequencies and sequencing costs are log-transformed for improved visibility of trends across a wide range of values. The y-axis for citations represents log10-transformed counts, where each unit increase corresponds to a tenfold increase in the number of citations. Similarly, the y-axis for sequencing costs reflects log10-transformed values, where each unit decrease corresponds to a tenfold reduction in the cost per megabase of sequencing. This transformation ensures that both very small and very large values are clearly represented, allowing for meaningful interpretation of exponential changes over time. The visualization emphasizes the rapid advancements in sequencing technology and the concurrent growth in research interest in multi-omics, personalized medicine, and GxE interactions

Genomics, the most established omics technologies, has profoundly enhanced our understanding of NCDs through extensive profiling of genetic variants such as SNPs, insertions-deletions, and structural variants [16, 24, 25, 16–[59]. Pioneering advancements in NGS technologies have been crucial, providing extensive genome-wide coverage that is faster and more cost-effective than ever before [60]. Significant and fast reduction in sequencing costs has spurred substantial growth in genomic and multi-omics research, making large-scale studies more feasible and affordable (Fig. 1). So far, over 6000 GWASs have been conducted for more than 3000 traits, yielding thousands of associated genetic variants [58, 59]. This represents a substantial advance over the pre-GWAS era when only a handful of genetic associations were robustly identified [58]. For instance, a GWAS of Crohn's disease implicated the IL-12/IL-23 pathway in the development of the disease, which subsequently informed clinical trials of drugs that targeted these pathways [61]. Furthermore, polygenic scores (PGSs) that aggregate genetic risk information across the genome are increasingly used to predict an individual's risk of developing NCDs and other diseases [61]. A recent clinical study demonstrated the effectiveness of PGS-based risk assessments for 10 NCDs, including coronary artery disease, atrial fibrillation, type 2 diabetes, chronic kidney disease, and breast cancer. Notably, this study returned genome-informed risk assessment results to patients, marking a significant milestone in clinical genetics [62]. Additionally, in psychiatric genomics, PGSs have shown promise in predicting treatment outcomes for mental health disorders, including treatment response, resistance, side effects, and hospitalization rates [63–[65].

Although GWASs have successfully identified replicable genetic variants associated with many NCDs and other traits, there are significant methodological and ethical challenges that must be addressed before these findings can be fully translated into preventive and clinical treatments. One major limitation is the poor transferability of findings across different genetic ancestries [48, 49]. This discrepancy largely stems from the underrepresentation of non-European ancestry in GWAS cohorts (only 14%) [48, 66]. This lack of diversity not only impedes the clinical application of PGSs but also exacerbates health disparities, because the benefits of ancestry-biased genetic research cannot equitably be distributed across populations [46, 47, 46–[69]. A critical aspect often overlooked is that Africa harbors the greatest human genetic diversity in the world, which offers unique opportunities for understanding genetic susceptibility to NCDs and other complex traits [70–[72]. The African Genome Variation Project (AGVP), for example, uncovered over 8 million novel variants, with a substantial proportion identified in Ethiopian and Zulu populations [73]. Moreover, African populations possess shorter haplotype blocks and complex population substructures, which allow for more precise fine mapping of disease susceptibility alleles [70, 71, 74]. This diversity, combined with the unique genetic adaptations in response to diverse climates, diets, and infectious diseases, underscores the necessity of expanding large-scale sequencing efforts in African populations. Incorporating these genomes will not only advance our understanding of NCDs but also ensure that the benefits of genomic medicine are equitably distributed across all populations [72].

Despite the slow progress, there are promising global efforts aimed at tackling the significant lack of diversity in genomic research. The Human Heredity and Health in Africa (H3Africa) initiative, the largest genomic research consortium in Africa, is spearheading this effort with a 10-year project aimed at studying the genetic basis of disease among African populations and establishing sustainable genomics research across the continent [66]. This initiative includes the creation of three biorepositories in Uganda, South Africa, and Nigeria, and the development of the Pan African Bioinformatics Network (H3ABioNet), supporting advances in handling biological data [66]. In Latin America, the Latin American Genomics Consortium is harmonizing data from existing cohorts and planning new recruitments to build a substantial biobank, addressing the underrepresentation of admixed populations [66]. In the United States, the All of Us Research Program aims to mirror the country's diversity by collecting data from over one million participants, half of whom are of non-European genetic ancestry. This program has identified over 275 million previously unreported genetic variants, with 77% of its participants coming from historically underrepresented communities in biomedical research [75]. Despite these encouraging efforts, the progress is far from sufficient. There is a substantial disparity within continents, particularly in Africa, Latin America, South Asia, and West Asia, where only a few countries have well-established biobanks [66]. This highlights the ongoing need for more comprehensive initiatives to ensure that genetic research benefits all global populations equitably.

Another limitation of standard population-based GWAS is the bias arising from population stratification and assortative mating, which can distort the estimated effects of variants on phenotypes [76–[79]. Standard-GWAS results are influenced by several factors, including the direct effects of alleles carried by an individual on their phenotype; the indirect effects of alleles carried by relative(s) through environmental influences (genetic nurture); and confounding due to population stratification and assortative mating. Although methods such as principal-component (PC) analysis and linear mixed models (LMMs) are used to adjust for population stratification, residual confounding often persists in GWAS summary statistics [78–[80]. These biases are particularly pronounced in polygenic scores (PGSs), which aggregate genetic risk information from thousands of variants [78]. Additionally, such biases can also impact post-GWAS analyses, including biological annotation, heritability estimation, genetic correlations, Mendelian Randomization (MR), and GxE interaction [78]. While family-based GWASs typically have lower power than population-based GWASs due to smaller sample sizes, they have been shown to mitigate biases from population stratification effectively [78, 80]. A recent within-family GWAS, conducted on a large sample of siblings, has demonstrated that within-family association estimates are significantly attenuated compared to standard GWAS estimates for traits such as depressive symptoms, height, and smoking [78]. The increasing availability of family-based data offers great potential for disentangling direct and indirect genetic effects affecting NCDs, thereby aiding in unraveling complex GxE interactions.

### Transcriptomics

Transcriptomics, through RNA sequencing (RNA-seq) technologies, has become instrumental in elucidating cellular pathways critical to the pathophysiology of many NCDs [81]. By analyzing all RNA transcripts, including coding and non-coding types, RNA-seq provides comprehensive insights into mRNA abundance, alternative splicing, nucleotide variations, and structural alterations [81, 82]. By revealing how gene expression is regulated and altered under various conditions, transcriptomics plays a pivotal role in bridging genotypic variations with phenotypic manifestations.

For instance, a study by Romanoski et al. (2010) integrated transcriptomics and genomics to examine human aortic endothelial cells' response to oxidized phospholipids, a key factor in atherosclerosis—a major cause of heart disease [83]. They treated cells with oxidized phospholipids, known to induce vascular inflammation, and simultaneously performed analysis to identify expression quantitative trait loci (eQTLs) influencing gene expression changes. This approach revealed that approximately one-third of the highly regulated transcripts exhibited gene-environment (GxE) interactions, often influenced by distal, trans-acting effects. Some notable interactions were further validated through small interfering RNA (siRNA) knockdown experiments, confirming the significant role of specific genetic loci in modulating gene expression responses to environmental stimuli [83]. This and other related studies [84–[86] illustrate how integrating genomic and transcriptomic data can uncover complex GxE interactions, enhancing our understanding of the genetic and environmental underpinnings of cardiovascular diseases and other NCDs.

Building on the capabilities of RNA sequencing technologies, the surge in transcriptomic data has prompted the establishment of comprehensive consortia tasked with managing, curating, and distributing these resources to the broader scientific community. Among the most noteworthy is The Cancer Genome Atlas (TCGA), which provides a rich repository of cancer-related transcriptomic data. Similarly, the Allen Human Brain Atlas offers specialized RNAseq databases focusing on brain diseases, encompassing studies on aging, dementia, and traumatic brain injury. Additionally, repositories such as the Gene Expression Omnibus (GEO), Encyclopedia of DNA Elements (ENCODE) [87], and the Genotype-Tissue Expression (GTEx [88]) Project significantly contribute to the availability of transcriptomic data across various tissues and conditions. By providing access to extensive transcriptomic data, these consortia support the ongoing exploration of how gene expression is intricately regulated and modified, thus continuing to bridge genotypic variations with phenotypic manifestations in complex disease research.

### Epigenomics

Epigenomics, which examines the full spectrum of epigenetic modifications such as DNA methylation and histone modification, plays a crucial role in understanding how environmental factors and genetic predispositions interact to influence the development of diseases [89]. These modifications regulate gene expression without altering the DNA sequence and are involved in critical processes like cellular differentiation and tumorigenesis [89, 90]. The epigenome's responsiveness to various environmental exposures—such as metals, air pollution, electromagnetic radiation—and lifestyle factors like diet, smoking, and physical activity, as well as the natural aging process, underscores its dynamic nature [89, 91]. For example, chronic exposure to arsenic and lead is associated with DNA methylation changes that heighten the risk of various cancers [92, 93]. Similarly, prenatal dietary factors like folate intake can alter the epigenome, influencing fetal development and disease susceptibility later in life [94–[96]. Additionally, medications such as sodium valproate (VPA), used for treating epilepsy and bipolar disorder, demonstrate the complexity of interactions between pharmacological treatments and epigenetic regulation by affecting gene expression through their histone deacetylase (HDAC) inhibitor properties [97, 98].

Epigenomics can provide a molecular framework to understand how GxE effects manifest in NCDs. Through studying epigenetic modifications, researchers can discover novel genes and pathways influenced by genetic factors and environmental exposures. The epigenome is partly regulated by the genome, with genetic variation influencing the establishment of DNA methylation marks [99–[102], while also being highly responsive to environmental factors [103–[105]. This dual regulation highlights the complexity of gene-environment interactions. For instance, the expression of certain NCD risk variants may depend on specific DNA methylation states, which environmental factors can alter. Alternatively, genetic variations might predispose certain epigenomic profiles to respond differently to environmental exposures, thus influencing disease risk [94]. Recent studies, such as those by Teh et al. (2014), have shown that a significant proportion of variably methylated regions, areas where methylation levels vary substantially among individuals, can be attributed to GxE interactions, revealing the intricate molecular mechanisms at play [106].

Advances in next-generation sequencing technologies have significantly enhanced the precision and scope of epigenic profiling [94]. While techniques like bisulfite conversion have been widely used to map methylation, they come with challenges, such as incomplete conversion and DNA degradation. The advent of long-read sequencing technologies, such as PacBio’s HiFi sequencing, has addressed some of these limitations. HiFi sequencing can directly detect 5mC methylation without the need for bisulfite conversion, offering both high accuracy and the ability to resolve methylation profiles alongside phased haplotyping in a single run [107]. This capability significantly improves our understanding of epigenetic modifications linked to genetic variants and environmental factors. On the other hand, major collaborative efforts like the NIH Roadmap Epigenomics project, the International Human Epigenome Consortium (IHEC), and ENCODE project have provided comprehensive maps of the human epigenome. By linking epigenetic changes to functional outcomes, these consortia enhance our understanding of the complex interactions that define health and disease, paving the way for advances in precision medicine.

### Proteomics

Proteomics explores the entire array of proteins produced or modified by an organism, offering crucial insights into the development and progression of NCDs. The proteome is highly dynamic, exhibiting considerable variability due to processes like alternative splicing, protein modifications, and the complex assembly of proteins into signaling networks [108]. These processes, regulated spatially and temporally, allow proteomics to measure critical changes in amino acid mutations, peptide isoforms, and posttranslational modifications (PTMs) [109, 110]. PTMs like phosphorylation, acetylation, and glycosylation are especially significant, as their dysregulation is often implicated in cancer, cardiovascular diseases, and neurodegenerative disorders [109, 110]. Proteomic profiles also capture responses to environmental stimuli, such as diet [111], chemical exposure [112], and smoking [113], highlighting their value in understanding complex gene-environment or proteome-environment interactions and refining the selection of target genes for further investigation [32].

A hallmark example of proteomics integration with genomic and phenotypic data is the UK Biobank Pharma Proteomics Project (UKB-PPP), a public–private partnership that profiled over 2,900 proteins in plasma samples from over 54,000 participants [114]. This initiative identified over 14,000 protein quantitative trait loci (pQTLs), with 81% being novel. By comparing results from different platforms like Olink and SomaScan and across diverse ancestries, the project underscored the power of multi-level data integration in revealing protein-level differences that influence disease studies, enhancing our understanding of genomic associations and disease mechanisms across populations [114]. Among the most notable associations with NCDs include a strong link between natriuretic peptide B (BNP) and heart failure and inflammatory bowel disease (IBD) associated with higher plasma levels of prostaglandin-H2 D-isomerase [114]. While most analyses focused on participants of European genetic ancestry (n = 34,557), ancestry-specific pQTL studies in African (n = 934), Central/South Asian (n = 920), and other non-European groups revealed unique variants, many of which were absent or rare in Europeans [115]. These findings underscore the importance of expanding proteomic studies to diverse ancestries to capture population-specific genetic and proteomic interactions, addressing disparities in disease risk and treatment. Another UK Biobank study identified over 5,000 associations between rare protein-coding variants and plasma protein abundances, significantly expanding our understanding of how rare variations influence proteomic profiles and highlighting their potential in identifying new therapeutic targets and biomarkers [116].

Despite its promise, proteomics faces challenges in scalability, cost and analytical complexity. High-throughput platforms such as mass spectrometry (MS) and proximity extension assays (PEA) enable precise protein profiling from minimal amounts of biological samples, but they remain costly, limiting their application in large-scale studies [117, 118]. The high dynamic range of protein expression and complexity of many PTMs and sequence variations pose further technical hurdles [109, 110]. Addressing these challenges is crucial for fully leveraging the potential of proteomics for novel biomarker discovery, targeted drug development, and understanding NCD mechanisms.

Public–private collaborations like the UKB-PPP highlight the transformative potential of proteomics to bridge the gap between genetics and phenotypes in multi-omics research. By integrating proteomics into population-scale biobanks, researchers can enhance causal gene identification, refine patient stratification, and accelerate therapeutic discovery. However, ensuring equitable applications requires broadening the representation of underrepresented populations and addressing cost barriers. These advancements will enable proteomics to significantly contribute to precision medicine and effective management of NCDs globally.

### Metabolomics

Metabolomics focuses on small-molecule metabolites—such as hormones, amino acids, and lipids—that serve as substrates, intermediates, and products of metabolism, offering a direct window into the biochemical pathways driving complex diseases, including NCDs [119]. Closer to the actual phenotype than mRNA or protein, metabolite levels provide a particularly valuable physiological readout because they integrate environmental and multiple regulatory inputs [120]. Each tissue or cell type has a unique metabolic signature, allowing metabolomics to highlight organ or tissue-specific changes linked to disease [120]. The dynamic nature of the metabolome, highly responsive to factors such as diet and chemical exposure, makes it indispensable for studying gene-environment interactions in NCDs [121]. Integrating metabolomics data with other omics layers, such as genomics and proteomics, enhances our ability to map metabolic pathways, predict metabolite abundances, and identify novel biomarkers and therapeutic targets across diverse populations.

Metabolomics enables the quantification of both endogenous metabolites and xenobiotics—foreign substances like environmental chemicals, pollutants, and drugs—offering a comprehensive view of how external exposures impact biological systems [119]. By analyzing these external compounds alongside changes in the endogenous metabolome, metabolomics reveals critical insights into the biological effects of environmental exposures. For example, a study on occupational exposure to trichloroethylene (TCE) identified TCE metabolites in human plasma and linked them to changes in endogenous metabolites associated with immunosuppression, hepatotoxicity, and nephrotoxicity, highlighting the toxic effects of TCE [122]. Similarly, the EXPOsOMICS project explored biofluids and exhaled breath for disinfection by-products (DBPs) from swimming pools, uncovering potential disruptions to metabolites in the tryptophan pathway [123]. In another study, researchers examined the relationship between SNPs in the methionine salvage enzyme APIP and mortality risk in sepsis triggered by infections like Salmonella. By analyzing plasma metabolomic profiles from about 1,000 patients, the study showed that sepsis survivors had significantly lower levels of the enzyme’s substrate, methylthioadenosine, than nonsurvivors, illustrating how genetic variation and metabolite levels jointly influence sepsis outcomes [124]. These examples demonstrate metabolomics’ capacity to unravel gene-environment interactions and the biological consequences of external exposures.

A key challenge in metabolomics is the identification and measurement of metabolites, but recent advancements have significantly eased this bottleneck [119, 120, 125]. Technological advances in nuclear magnetic resonance (NMR) and mass spectrometry (MS)-based methods, such as GC–MS and LC–MS, have also improved the precision and range of metabolite quantification. Expanded metabolite databases, such as The Human Metabolome Database (HMDB) and XCMS-METLIN, now contain tens of thousands of metabolites, including xenobiotics from environmental sources. Additionally, various bioinformatics tools now enable more robust analysis, linking metabolic signatures to disease states and outcomes, thus enhancing the potential of metabolomics in NCD research [119].

### Exposomics

**Fig. 2:**
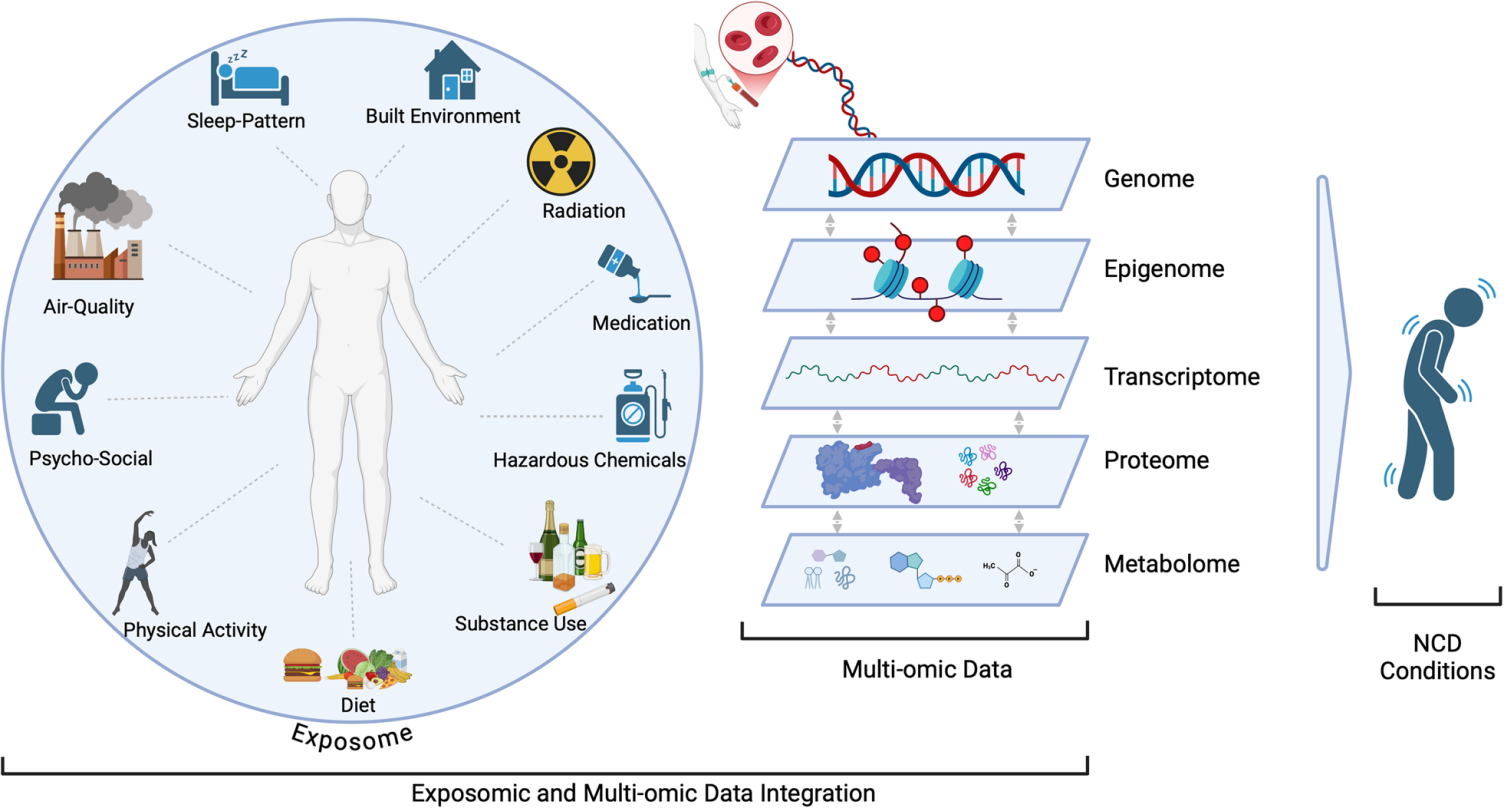
The Exposome and Multi-Omic Interactions for GxE Interaction Analyses in the Context of NCDs. The exposome encompasses the cumulative impact of environmental influences, including external factors (e.g., urban and built environments, air and water quality, soil contaminants such as heavy metals and persistent organic pollutants), lifestyle factors (e.g., physical activity, diet, smoking, alcohol use, and sleep patterns), psychosocial factors, and social and economic determinants (e.g., socio-economic status, access to resources). It also includes occupation and residential exposures (e.g., noise pollution and indoor air quality), as well as chemical and physical agents. These diverse influences interact with multiple biological layers—genome, epigenome, transcriptome, proteome, and metabolome—triggering complex, nonlinear responses. These interactions drive the onset, progression, and development of NCDs, including cardiovascular diseases, diabetes, respiratory illnesses, and cancers. Integrating the exposome into multi-omic studies is essential to unraveling these dynamics and advancing precision medicine approaches for chronic disease prevention and management. NCD—non-communicable diseases. Schematic plot created using BioRender (https://BioRender.com)

**Fig. 3:**
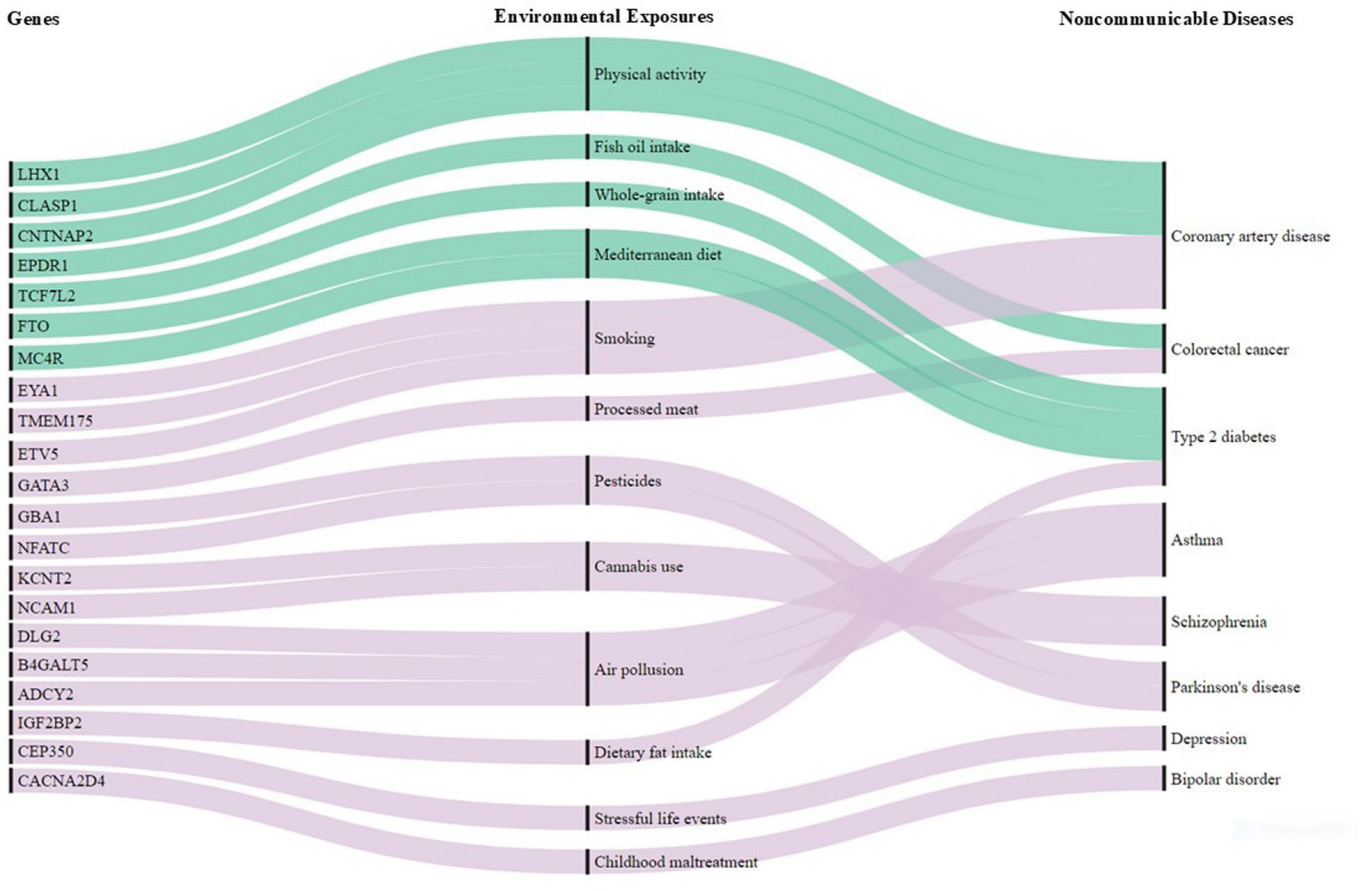
Notable examples of how genetic and environmental factors interact to influence the risk of developing NCDs. This Sankey diagram illustrates examples of gene-environment interactions in non-communicable diseases (NCDs) identified through genome-wide gene-environment interaction and Mendelian randomization studies; it is not an exhaustive list of GxE interaction studies in NCDs. Genes (left vertical lines) are connected through environmental exposures (middle vertical lines) to NCDs (right vertical lines); green lines represent interactions that reduce NCD risk, while light purple lines indicate increased risk. Studies using candidate-gene approaches were excluded due to inherent limitations such as high risk of false positives, lower replication rates, selection bias, and limited genetic coverage. A full list of the SNPs (rsID and PMID) implicated in the GxE interactions and a brief description of each GxE interaction are shown in Table S1

Exposomics is a burgeoning field that explores the comprehensive impact of environmental factors on human health over an individual's lifetime [126]. This discipline considers various exposures—from chemical and biological agents to psychosocial factors, socioeconomic status and interpersonal relationships. These factors can trigger various biological responses, including changes in gene and protein expression, which in turn may influence the microbiome and epigenome [126]. This complex interplay underscores how environmental factors, intertwined with genetic predispositions, contribute to the development of NCDs (Fig. 2). Examples of replicated gene-environment interactions include *BRCA-1* associated protein-1 (BAP1) mutations and asbestos exposure for mesothelioma [127], chromodomain helicase DNA-binding protein 8 (*CHD8*) and pesticide exposure for autism spectrum disorder [128], the fat mass and obesity-associated gene (*FTO*) and physical activity for obesity, and dopamine receptor D4 (*DRD4*) and parenting style for attention-deficit/hyperactivity disorder (ADHD) [129]. These interactions highlight how specific genetic susceptibilities can be activated or exacerbated by environmental factors, demonstrating the crucial role of exposomics in understanding NCDs. A subset of notable GxE interactions implicated in common NCDs is shown in Fig. 3. While most of these GxE examples have focused on single environmental variables, the broader and more systematic measurement of environmental factors—such as those captured through exposomics—holds tremendous potential for deepening our understanding of complex diseases. Although still in its early stages, a few studies have ventured into multi-exposure genome-wide interaction analysis, jointly modeling the effects of the genome and the environment using methods like StructLMM (structured linear mixed model) [130], GxEMMs (GxE Mixed Model) [131], and IGE (integrative analysis of genomic and exposomic data) [132]. These approaches account for genome-exposome correlations and the interrelationships among exposome variables, offering a more holistic view of gene-environment interactions [133]. However, such methods are often computationally intensive and challenging to interpret, underscoring the complexity and potential of exposomics in advancing our understanding of NCDs.

Recent technological advancements, especially in high-resolution mass spectrometry (HRMS) and wearable devices, have significantly improved the ability to measure the exposome with precision and individual specificity [134]. HRMS enables the detailed detection of small molecules in biological samples like plasma and urine, allowing for an in-depth analysis of exposures to pharmaceuticals, pollutants, and nutrients [135]. Complementing this, wearable technologies such as silicone wristbands and other personal passive samplers have emerged as powerful tools for capturing personal exposure data. These devices can monitor environmental exposures in real-time across different settings and critical life stages, such as during pregnancy or early childhood, thus providing a dynamic and personalized exposome profile [136]. For instance, studies employing wristband samplers in China have successfully profiled personal chemical exposures, demonstrating the diversity and complexity of environmental interactions individuals face daily [137]. Another innovative approach uses a miniaturized wearable device that samples air to capture particulates, further analyzed using HRMS to identify both hydrophobic and hydrophilic chemical compounds [138]. These studies exemplify how wearables can offer insights into the spatiotemporal dynamics of personal exposures and their potential health impacts. By integrating data from these wearables with systems biology approaches, researchers can now begin to unravel the intricate gene-environment interactions that significantly influence the pathogenesis of NCDs, paving the way for targeted prevention and therapeutic strategies.

Furthermore, new initiatives are expanding the scope of exposomics research, leveraging large-scale resources to deepen our understanding of environmental contributions to health. The All of Us Research Program [139] is increasingly integrating environmental exposure data with genomic, clinical, and demographic information from its diverse cohort of participants. By linking geospatial data with exposure estimates from tools like the Environmental Justice Index, the program aims to examine how environmental factors influence disease susceptibility. Similarly, the European Human Exposome Network (EHEN), the world’s largest network of exposome-focused projects, is advancing research on the health impacts of air pollution, noise, chemicals, and urbanization [140]. Together, these efforts are equipping researchers with unparalleled data resources to elucidate the complex interplay between genetic and environmental factors in NCDs (Fig. 3).

## Electronic health records (EHRs)

EHRs are digital versions of patients' medical histories, encompassing a broad spectrum of data, including demographics, medical histories, vital signs, laboratory test results, radiology images, diagnoses, treatment procedures, and medications [37]. Longitudinal data available in practice-based EHRs, such as those from chronic disease management clinics, enable researchers to characterize genetic factors with small but reproducible effects on drug outcomes. For instance, the electronic Medical Records and Genomics (eMERGE) network, supported by EHRs, presents a novel opportunity to coordinate such investigative efforts across multiple institutions, facilitating the dissection of GxE interactions.

With advancements in healthcare technology, EHRs have expanded to include Personal Health Records (PHRs), which capture out-of-clinic data such as daily behaviors and physiological measurements collected by smart wearable devices. In the realm of precision medicine, EHRs serve as crucial repositories that connect detailed clinical data with genetic profiles from multi-omics studies. This integration offers a holistic view of a patient's health landscape, combining structured data like lab results and diagnosis codes with unstructured data, such as free-text clinical notes. Although rich with information, EHRs present challenges in data heterogeneity, quality, and management, especially given their mix of unstructured and structured formats [37]. These challenges complicate the extraction and analysis of data but are essential to address for leveraging EHRs in enhancing our understanding of NCDs through multi-omics integration.

## Advances in multi-omics integration techniques

The field of multi-omics integration has rapidly evolved to enhance our understanding of the GxE interactions that underlie NCDs and other complex diseases [32]. Individual omics approaches, such as GWAS, have successfully identified numerous SNPs associated with various NCDs [14–[16, 141]. However, the challenge of uncovering the functional roles of these SNPs, especially those located in non-coding regions, necessitates the integration of genomic data with transcriptomic, proteomic, metabolomic, epigenomic, and other omics datasets [32]. This comprehensive integration is crucial for mapping the flow of genomic information and elucidating the interactive networks essential for the onset and progression of NCDs.

New advances in analytical methods and software have significantly improved our ability to integrate data across multiple omics layers, offering a deeper understanding of how genetic variations interact with environmental factors to influence biological pathways and disease outcomes [41, 44, 60, 81]. By synthesizing data from various domains, multi-omics approaches provide powerful tools for elucidating the intricate dynamics of GxE interactions in NCD research, paving the way for targeted interventions and personalized medicine. These integration techniques utilize approaches that often fall under two broad categories: a suite of post-GWAS analyses and machine learning-based methods [35]. Post-GWAS analysis enhances the interpretation of GWAS results by incorporating additional omics data, enriching our understanding of how identified genetic variants influence disease phenotypes. Conversely, machine learning methods utilize algorithms to model complex interactions across different biological layers, offering robust tools for deciphering the intricate dynamics of GxE interactions and advancing NCD research.

### Post-GWAS multi-omics integration approaches

#### Enrichment-based methods

Enrichment-based methods provide a powerful way to integrate GWAS data with additional omics layers, thereby enhancing the understanding of complex GxE interactions that underpin NCDs [24]. These approaches utilize overlap, correlation, or association analysis techniques to identify quantitative trait loci (QTLs) that are significantly associated with molecular features such as gene expression (eQTLs), methylation intensity (meQTLs), and protein levels (pQTLs) [142]. For example, the GTEx [88], ENCODE [87], and Roadmap Epigenomics [143] projects have systematically cataloged associations between SNPs and various molecular features, creating valuable research resources. Integration of GWAS significant variants and QTLs is achieved through overlapping or positional mapping with functional annotations, confirmed by statistical tests to ensure enrichment is significant and not due to random chance.

For instance, a study [144] on atrial fibrillation utilized an integrative multi-omics approach combining genomics, transcriptomics, and proteomics from human atrial tissues. This cross-sectional study identified the widespread effects of genetic variants on both mRNA and protein expression, pinpointing transcription factor NKX2-5 as a crucial link between a GWAS SNP and atrial fibrillation [144]. Similarly, in schizophrenia, enrichment-based methods revealed that risk loci were associated with meQTLs in fetal brain tissue (most notable associations include, rs2535627-cg11645453 and rs4648845-cg02275930), suggesting that altered DNA methylation may play a role in the disease's pathogenesis [145]. These and numerous other examples illustrate how enrichment methods can reveal the cellular origins and molecular networks of disease mechanisms.

However, these enrichment estimates can be biased by factors like linkage disequilibrium and the presence of multiple functional variants [146]. Advanced statistical methods, such as hierarchical Bayesian modeling [147] and permutation tests [146], are employed to mitigate these biases. These strategies not only aid the functional annotation of genetic data but also the discovery of novel biomarkers, offering insights into the tissues and mechanistic pathways involved in NCDs.

#### Statistical fine-mapping methods

Statistical fine-mapping methods crucially enhance the integration of GWAS with various quantitative trait loci (QTLs), aiding in identifying causal variants that may influence NCDs and other complex conditions [148]. These approaches, such as colocalization and Mendelian randomization (MR), are pivotal in determining the specific genetic variants that could contribute to both observed molecular traits and disease phenotypes. Colocalization analysis, often conducted using Bayesian statistical methods [149, 150] among others, evaluates whether a genetic variant (s) can be linked to both a GWAS trait and a molecular QTL. This can highlight potential causal genes and pathways implicated in diseases, exemplified by research in Alzheimer’s disease that linked genetic risk variants with eQTLs affecting novel and known genes [151].

Mendelian randomization uses genetic variants as instrumental variables to explore causal relationships between modifiable molecular traits and disease outcomes, functioning under the strong assumption that the variant influences the disease solely through its effect on an intermediary molecular trait—a premise that is challenging to validate [152–[154]. For example, a study on depression linked genetically regulated brain protein levels to the disease, suggesting causality through MR analysis [155]. Furthermore, the comprehensive review by Markozannes et al. (2022) on cancer risk used MR to validate causal associations, such as the effects of BMI on kidney and endometrial cancers and circulating sex hormones on breast cancer [156]. These robust associations highlight the utility of MR in confirming causal pathways, providing a basis for targeted preventive strategies and therapies. Both colocalization and MR provide insights into potential causal mechanisms, with significant colocalization often implying a causal pathway that might be validated through MR. These methods not only pinpoint underlying genetic interactions but also guide the development of targeted therapies and preventive measures across a spectrum of complex diseases.

Recent methodological advances in multi-ancestry fine-mapping strategies have significantly enhanced the ability to identify causal genes, offering insights beyond those provided by single-ancestry approaches [157, 158]. MA-FOCUS (multi-ancestry fine-mapping of causal gene sets), for example, integrates GWAS, eQTL, and LD data from multiple ancestries without assuming shared eQTL architecture [158]. It focuses on consistency in causal genes across populations, improving accuracy in identifying disease-relevant genes for traits like hematopoietic and cardiovascular diseases. Similarly, SuSiEx, building on the single-population framework of Sum of Single Effects (SuSiE), offers a powerful cross-population fine-mapping tool. It integrates data across ancestries, models population-specific allele frequencies and LD patterns, and handles multiple causal variants within genomic regions using GWAS summary statistics [157]. In evaluations involving traits from the UK Biobank and Taiwan Biobank, and a schizophrenia GWAS across East Asian and European ancestries, SuSiEx fine-mapped more association signals, produced smaller credible sets, and achieved higher posterior inclusion probability for causal variants, even capturing population-specific causal variants [157]. Both MA-FOCUS and SuSiEx highlight the critical importance of including genetic data from diverse ancestries to improve the resolution of genetic studies and to uncover more precise therapeutic targets.

#### Imputation-based methods

Imputation-based methods are a powerful tool for integrating genomic and multi-omics data, utilizing extensive datasets from sources such as GTEx [88] and ENCODE [87]. These methods depend on a reference panel built from robust genetic prediction models derived from genotype data and molecular measurements (e.g., gene or protein levels) of healthy individuals [35]. These models are crafted using statistical techniques, including LASSO, ridge regression, and elastic net [35]. Through this approach, imputation-based methods impute molecular features within GWAS datasets, enabling the identification of associations between genetically predicted molecular features and various NCDs. Key findings from this method often reveal molecular features that are differentially expressed between cases and controls, thus highlighting potential pathways of disease manifestation. Transcriptome-wide association studies (TWAS) are a common application, successfully identifying molecular features linked to various traits and conditions [159].

Beyond gene expression, imputation-based integration has been adapted to explore other molecular features, such as DNA methylation and protein levels, though these remain less commonly applied compared to TWAS [159]. Furthermore, multi-omics integration approaches often extend beyond the use of GWAS data alone, employing previously described enrichment-based methods to merge findings from different omic layers—such as transcriptomics and epigenomics—through overlap and correlation analyses. These integrated analyses provide deeper insight into the complex interactions during the pathogenesis of NCDs. For instance, in one integrative study, researchers analyzed the relationships among intestinal microbiota, serum metabolome, and inflammatory cytokines in groups with and without schizophrenia [160]. Utilizing weighted gene co-expression network analysis [161], they identified significant co-abundance clusters of metabolites and gut bacteria, which correlated with cytokine levels. This suggests that specific bacteria could influence inflammatory responses through metabolic modulation [160]. Such integrative studies underscore the potential of using multi-omics data to uncover biological networks involved in NCDs, particularly highlighting pathways such as the gut-brain and gut-immune axes, which are crucial for understanding complex diseases (Additional file 1).

### AI/machine-learning-based method

Machine learning (ML) methods are increasingly used to understand the complex GxE interactions in various NCDs [28, 32, 162, 163]. These methods adeptly handle the integration of noisy, high-dimensional multi-omic datasets, essential for elucidating the multifaceted biological processes underlying NCDs [162, 163]. Several integration strategies have been developed, each tailored to optimize the handling of these complex datasets in different scenarios [37]. Early integration, for example, concatenates datasets sample-wise, creating a comprehensive input matrix for ML models [164, 165]. However, the sheer size and complexity of such integrated data can be challenging for many ML algorithms, especially with smaller sample sizes. To mitigate these challenges, other strategies such as mixed integration [166]—which reduces dataset complexity individually—and intermediate integration—which reduces complexity jointly—are utilized [167–[169]. Late integration, conversely, analyzes each omics dataset independently before aggregating the outputs for a final decision [170]. In contrast, hierarchical integration systematically incorporates known biological regulatory frameworks into the analysis, reflecting the sequences of molecular interactions [171].

The versatility of ML methods facilitates a broad spectrum of applications in multi-omics data integration for NCDs, from diagnostic classification to prognosis prediction and evaluating treatment responses. The forthcoming sections will explore these applications in detail, presenting examples of specific ML frameworks that have shown promise in enhancing our understanding of many NCDs and other complex diseases. By leveraging these advanced ML approaches, researchers can pinpoint potential biomarkers, unravel disease mechanisms, and enhance the personalization of healthcare—key components in advancing the field of precision medicine for chronic diseases. In Additional file 2: Table S2, we provide a non-exhaustive list of multi-omics integration and GxE interaction analyses approaches.

#### Diagnostic classification

Diagnostic classification through ML involves accurately grouping patients into predefined classes representing specific disease diagnoses, a process crucial for managing NCDs [162]. In cardiovascular disease (CVD), ML classifiers have been utilized to predict CVD and related risk factors from omics data, illustrating the application's potential. A study by Drouard et al. (2024) compared various ML strategies using blood-derived metabolomics, epigenetics, and transcriptomics data to predict CVD risk factors [172]. The findings revealed that multi-omics predictions generally outperformed single-omics predictions, particularly in distinguishing individuals with extreme levels of CVD risk factors. Techniques like semi-supervised autoencoders, which refine feature representation before classification, demonstrated improved predictive accuracy over unsupervised methods, highlighting the capabilities of ML to enhance diagnostic precision in complex disease settings [172].

In the realm of cancer diagnostics, ML has shown significant promise. For instance, a study by Khadirnaikar et al. (2023) on non-small cell lung cancer (NSCLC) employed ML to identify novel subtypes, enhancing prognostic accuracy and treatment personalization [173]. By applying consensus K-means clustering to multi-omics data, the study identified five distinct NSCLC clusters with varying survival outcomes and genetic characteristics, demonstrating the superior performance of multi-omics over single-omics models. Similarly, a novel approach by Abassi et al. (2024) utilized a combination of ML and deep learning (DL) techniques to improve diagnostic accuracy for leukemia [174]. Employing various ML algorithms and deep learning networks like recurrent neural networks (RNNs), they achieved up to 98% accuracy in predicting leukemia from multi-omics data. This approach not only emphasizes the potential of integrating various omics data for cancer diagnostics but also showcases the efficiency of ML and DL in refining diagnostic classifications across different cancer types (Additional file 3).

Similarly, in psychiatric disorders, which share overlapping genetic, environmental risk factors, and symptomatology, ML tools are invaluable for refining diagnosis. Xie et al. (2021) demonstrated this by using gene expression and DNA methylation data to construct models that effectively distinguished patients with major depressive disorder (MDD) from healthy controls [175, 176]. Their approach identified genes that were either upregulated and hypomethylated or downregulated and hypermethylated in MDD patients. Although the gene expression classifier exhibited superior predictive power compared to the DNA methylation classifier, both models underscore the potential of ML in enhancing diagnostic accuracy.

#### Clinical outcome (Risk) prediction

Risk prediction is a vital ML application in the multi-omics analysis of NCDs [35]. This approach leverages ML to identify and prioritize molecular features that may forecast an increased risk of diseases. Typically, these features are unearthed through detailed single-omics analyses or ML-based feature selection within advanced integration strategies. Once identified, these molecular characteristics inform the development of statistical models designed to predict individual disease risks.

A notable method employed in risk prediction is the generation of polygenic risk scores (PGS), which calculate an individual's disease susceptibility based on quantitative trait loci (QTLs) and other regulatory genetic variants [24, 58]. These scores sum up an individual's risk alleles, each weighted by its effect size derived from GWAS. Techniques such as penalized regression—LASSO, elastic net, ridge regression—and Bayesian methods refine these risk scores, enhancing their predictive accuracy. An innovative adaptation in this domain involves integrating PGS with other omics data, which allows for a more nuanced interpretation of genetic contributions to disease risk [48, 58].

For instance, in a study by Wang et al. (2022), researchers explored the integration of multi-omics data for predicting clinical outcomes in neuroblastoma, a complex cancer [177]. They employed network-based methods, constructing Patient Similarity Networks (PSN) by assessing distances among patients using omics-derived features. Two distinct integration strategies were tested: network-level fusion, using the Similarity Network Fusion algorithm to merge PSNs across various omics types, and feature-level fusion, combining network features from individual PSNs [177]. Their findings highlighted that network-level fusion provided superior performance in integrating diverse omics data, demonstrating the potential of ML to enhance outcome predictions in NCDs through multi-omics integration techniques. Despite these advances, the clinical adoption of such models remains modest, underscoring the ongoing challenges in model validation and generalizability within healthcare settings.

#### Treatment response prediction

Predicting treatment responses is another critical application of ML in the context of multi-omics for NCDs [35]. This ML application spans various treatment-response assessment regimes, including pharmacotherapy, psychotherapy, and more, aiming to forecast outcomes such as prognosis, relapse, or therapeutic efficacy [178, 179]. Particularly in chronic diseases where treatment paths can vary significantly among individuals, leveraging multi-omics data can markedly enhance the precision of these predictions.

In cancer, where genetic heterogeneity strongly influences treatment outcomes, ML models have shown promise in predicting responses to anticancer drugs. A study by Wang et al. (2022) illustrates this with a deep neural network that integrates multi-omics data—including gene expressions, copy number variations, gene mutations, protein expressions, and metabolomics—from cancer cell lines [177]. The model features innovative components such as a graph embedding layer to incorporate interactome data and an attention layer to prioritize relevant omics features. This approach achieved an impressive R^2^ value of 0.90, outperforming standard neural networks in predicting drug responses using data from the Cancer Cell Line Encyclopedia (CCLE) and the Genomics of Drug Sensitivity in Cancer (GDSC). This example underscores the power of ML in harnessing multi-omics data to enhance the personalization of cancer treatments.

Another study by Joyce et al. (2021) explored the predictive power of combining genomics and plasma metabolomics to determine the effectiveness of combination pharmacotherapy in treating major depressive disorder (MDD) [180]. They developed two models: one using only metabolomics and another incorporating both metabolomics and genomics. The latter, a multi-omics approach, utilized penalized linear regression and XGBoost algorithms, demonstrating superior predictive performance as evidenced by a higher area under the curve (AUC) compared to the metabolomics-only model. This study underscores the added value of integrating multiple types of omics data to enhance the accuracy of predicting treatment responses.

#### Estimating GxE interactions

Unraveling gene-environment (GxE) interactions is crucial for understanding the complex etiology of NCDs. However, analytical tools for GxE interaction analysis remain limited due to challenges posed by high data dimensionality, significant noise, and heterogeneity in genetic and environmental factors across populations, which can obscure true interactions and hinder replicability. Traditional GxE interaction analyses often rely on regression techniques [181–[184], linking response variables to main genetic and environmental effects and their interactions. These methods face limitations such as stringent requirements to maintain a "main effects, interactions" hierarchy [183]. This hierarchy demands that if an interaction effect is identified, its corresponding main effects must also be considered in the model, which complicates the analysis by imposing additional constraints on variable selection [185, 186]. Moreover, high dimensionality demands multiple comparison adjustments, increasing the risk of Type II errors (failing to detect true effects), and many studies lack sufficient power due to small effect sizes and limited sample sizes [187].

The emergence of large-scale biobanks and observational studies like the UK Biobank [188], the All of Us Research Program [139], FinnGen [189], and initiatives supported by the Barcelona Institute for Global Health (ISGlobal) are helping address sample size limitations by providing extensive genetic and environmental data across diverse populations. Leveraging these rich datasets, researchers have turned to machine learning (ML) and artificial intelligence (AI) approaches to enhance GxE interaction analysis [190]. For instance, Wu et al. (2023) recently developed a novel methodology that leverages deep learning to enhance GxE interaction analysis [191]. This approach integrates deep neural networks with penalization strategies to simultaneously estimate and select significant GxE interactions and corresponding main effects while respecting the required hierarchical structure. Demonstrations through simulation studies and applications in NCD contexts, such as lung adenocarcinoma and skin cutaneous melanoma, show that this method not only manages the complexity of the data but also surpasses traditional regression methods in predictive accuracy and feature selection [191].

Madhukar et al. (2019) also introduced BANDIT, a Bayesian machine-learning approach for drug target identification using diverse data types [192]. BANDIT integrates over 20 million data points from six distinct data types – including drug efficacies, transcriptional responses, drug structures, adverse effects, bioassay results, and known targets – to predict drug-target interactions. Benchmarking showed approximately 90 percent accuracy in correctly identifying known drug targets across over 2,000 small molecules. Applied to compounds without known targets, BANDIT generated novel molecule-target predictions that were experimentally validated, including identifying new microtubule inhibitors effective against resistant cancer cells [192]. Although primarily focused on drug discovery, BANDIT exemplifies how integrating heterogeneous omics data through machine learning can elucidate complex biological interactions, including GxE interactions relevant to NCDs.

Similarly, other ML-based methods have shown promise in addressing the complexities of GxE interactions [193]. Zou et al. (2010) introduced a nonparametric Bayesian approach for mapping quantitative trait loci (QTL) that captures both main effects and higher-order interactions, including gene-environment interactions, without requiring explicit specification of interaction terms [194]. This method employs a Gaussian process prior combined with variable selection to identify important genetic and environmental factors. By modeling all potential interactions in a single framework, it avoids the computational and multiple-testing challenges associated with parametric approaches. Applied to the polygenic mouse model of obesity, the method identified key quantitative trait loci (QTLs) influencing fat pad weight and highlighted how nonparametric Bayesian variable selection could improve the detection of GxE interactions in complex traits.

Spanbauer et al. (2020) employed a non-parametric machine learning approach using Bayesian additive regression trees with mixed models (mixedBART) for precision medicine. This method adeptly identifies patient characteristics associated with treatment effect heterogeneity in clinical trials [195]. In a study focusing on type II diabetes mellitus among African-American patients, mixedBART predicted individualized treatment effects based on demographic and health measures. While additional analyses showed insufficient evidence for treatment effects, mixedBART facilitated the multi- exploration of treatment heterogeneity, underscoring its potential in GxE interaction studies [195].

In addition, the advent of multimodal medical large language models (LLMs) offers promising avenues for future GxE interaction studies in NCDs. Building on established medical LLMs [196, 197], several multimodal models such as LLaVA-Med (Large Language and Vision Assistant for BioMedicine) have been proposed [198]. These models are designed to process medical images and generate text-based interpretations, demonstrating medical image understanding and diagnosis capabilities. While current multimodal LLMs primarily handle modalities like text and imaging data, there is growing interest in extending these models to incorporate molecular-level omics data, including genomics. For instance, preliminary efforts like MedGPT have explored analyzing genomic data using LLMs, although they remain at a proof-of-concept stage with preliminary results [199]. As these models evolve and integrate more diverse datasets, they have the potential to enhance our ability to interpret complex biological interactions, including GxE interactions relevant to NCDs. However, significant challenges remain, and more research is needed to fully realize the integration of multimodal omics data in LLMs.

**Fig. 4:**
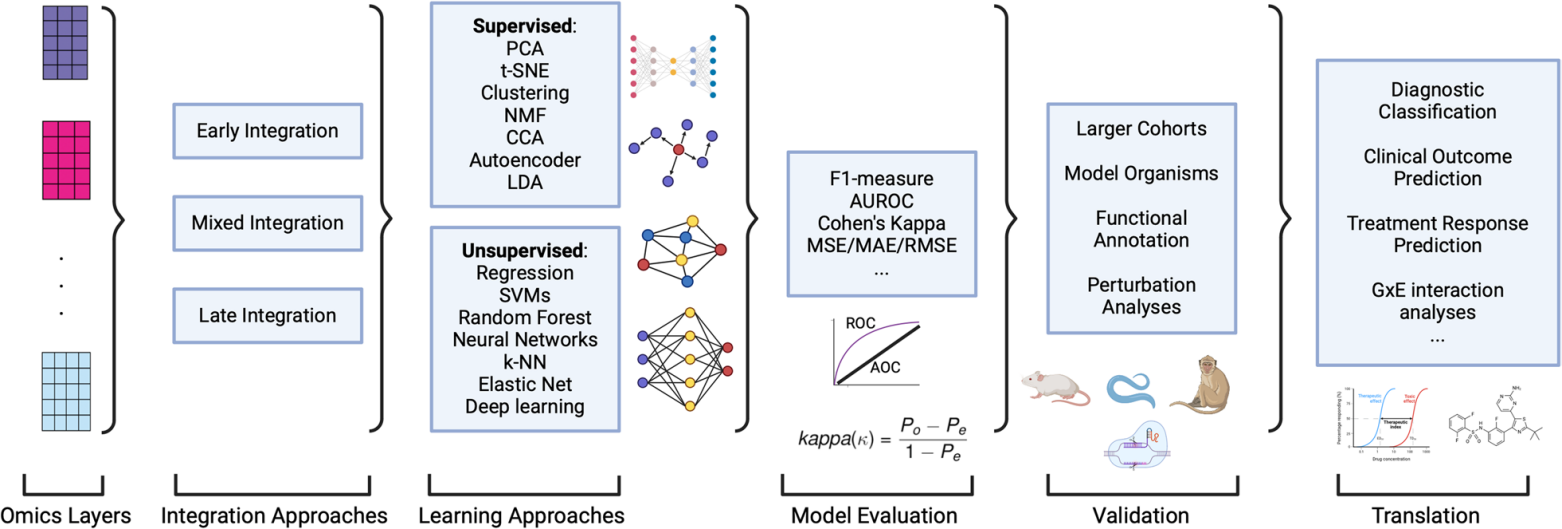
Schematic Overview of AI/ML-based Multi-Omics Data Integration Workflow. This schematic illustrates a simplified workflow for multi-omics data integration, highlighting key steps in processing, analyzing, and translating multi-omics datasets. The process begins with omics layers (e.g., genomics, transcriptomics, proteomics, metabolomics), integrated using approaches like early integration (merging raw data), mixed integration (combining intermediate features), and late integration (aggregating model outputs). These datasets are analyzed using unsupervised learning methods, including Principal Component Analysis (PCA), t-Distributed Stochastic Neighbor Embedding (t-SNE), clustering, Non-Negative Matrix Factorization (NMF), Canonical Correlation Analysis (CCA), autoencoders, and Latent Dirichlet Allocation (LDA), as well as supervised methods like regression, Support Vector Machines (SVMs), Random Forests, Neural Networks, k-Nearest Neighbors (k-NN), Elastic Net, and deep learning. Model performance is evaluated using metrics such as F-measure, Area Under the Receiver Operating Characteristic Curve (AUROC), Cohen’s Kappa, and error measures like Mean Squared Error (MSE), Mean Absolute Error (MAE), and Root Mean Squared Error (RMSE). Validation ensures robustness and biological relevance through larger cohorts, model organisms, functional annotation, and perturbation analyses. Finally, insights are translated into diagnostic classification, clinical outcome prediction, treatment response prediction, and gene-environment (GxE) interaction analysis. This schematic is not exhaustive but provides a simplified guide to navigate the manuscript’s discussion on multi-omics data integration

In summary, these advancements illustrate the growing role of ML/AI tools in addressing the challenges of GxE interaction analysis in NCDs and other complex diseases. By harnessing large and diverse datasets and employing sophisticated analytical methods, researchers can better understand the complex interplay between multi-omic factors and the exposome. However, applying AI/ML methods in this context also presents challenges. Bias remains a significant concern, as algorithms trained on datasets that underrepresent certain demographic groups can yield skewed predictions, potentially exacerbating existing health disparities among populations affected by NCDs [200]. The “black box” nature of many AI/ML models, particularly deep learning approaches, poses another hurdle, as the lack of interpretability may undermine clinical decision-making and trust, especially when transparent reasoning is crucial for evaluating risk factors or treatment options [201]. Furthermore, the use of sensitive patient data in NCD research heightens the risk of privacy breaches, raising complex ethical and legal challenges in data governance [202]. Overcoming these challenges requires diverse and representative training datasets, the development of interpretable AI models tailored to NCD applications, and robust privacy protections to ensure ethical and equitable use of AI/ML in advancing GxE research and clinical practice. Together, these efforts not only enhance our understanding of disease mechanisms but also contribute to the development of personalized interventions and treatments (Fig. 4).

### Current challenges and opportunities

#### Diversity of omics and multi-omics datasets

**Fig. 5:**
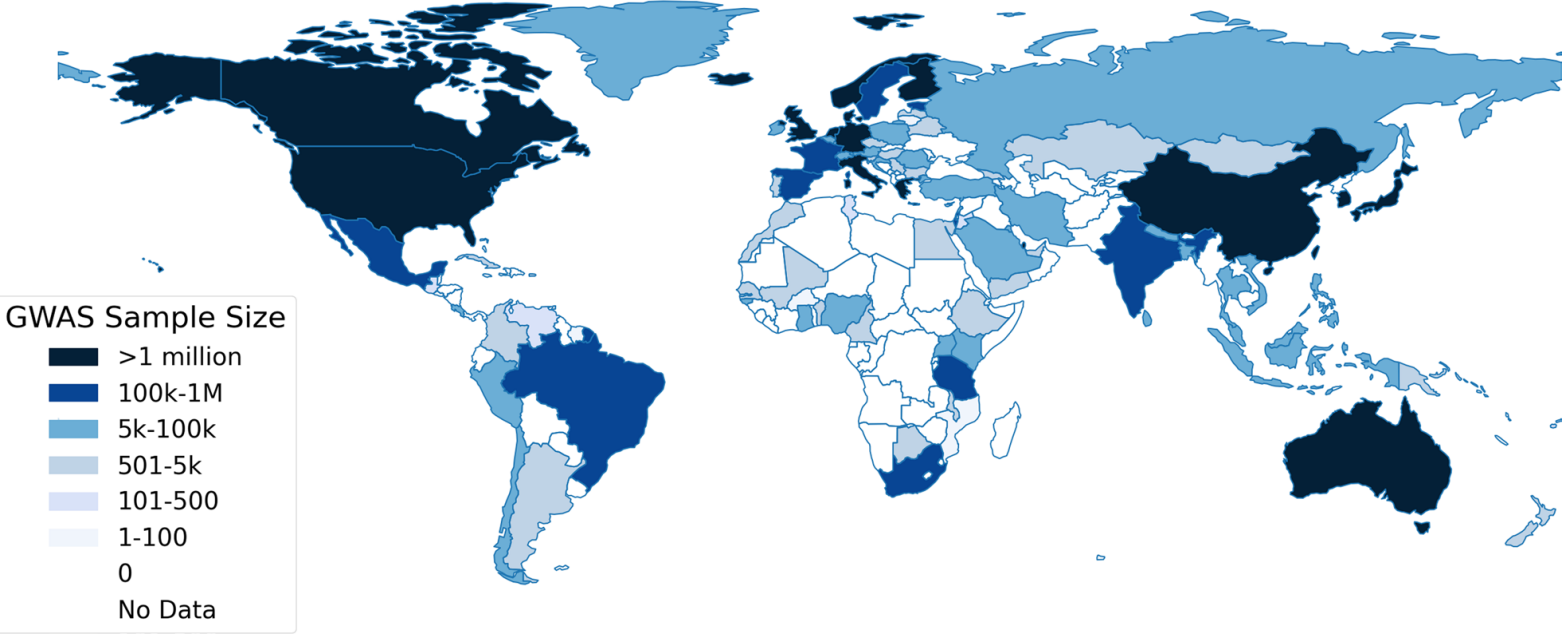
Global Distribution of Total GWAS Sample Sizes by Country. This map illustrates the geographic distribution of cumulative participants in genome-wide association studies (GWAS) across various countries for years where data are available, highlighting global disparities in genetic research participation. Data were sourced from Mills, M.C., and Rahal, C. (2020) in their study "The GWAS Diversity Monitor Tracks Diversity by Disease in real-time," published in Nature Genetics, 52, pp. 242–243, https://doi.org/10.1038/s41588-020-0580-y. The Leverhulme Centre for Demographic Science maintains the data

Despite efforts to diversify genomic datasets, the vast majority of GWAS, about 85% as of 2023, predominantly feature individuals of European genetic ancestry [203]. Progress toward including under-represented populations has been slow, with the share of studies involving these groups either stagnating or even declining in recent years [48]. Although there has been a modest rise in the representation of Asian ancestries, African, Latin American, and Indigenous populations remain markedly underrepresented [66]. This imbalance is compounded by the over-reliance on easily accessible and homogeneous resources like the UK Biobank, which primarily comprises individuals of European ancestry, whereas other ancestry groups often have limited data repositories available [66]. Figure 5 presents the global distribution of total GWAS sample sizes by country, underscoring significant regional disparities.

This lack of diversity leads to a substantial problem: PGSs derived from predominantly European datasets show dramatically reduced predictive accuracy when applied to non-European populations [48, 49]. For instance, Martin et al. (2019) reported a decline in PGS accuracy of about 37%, 50%, and 78% for individuals of South Asian, East Asian, and African ancestries, respectively [48]. Further studies, such as those by Privé et al. (2021) and Ding et al. (2023), confirm that PGS accuracy not only diminishes across different ancestries but also varies significantly within them depending on the genetic distance from the European training populations [49, 204]. The limited generalizability of these genetic insights could potentially exacerbate health disparities, underscoring the urgent need to broaden the genetic diversity in omics research to ensure that genomic advancements benefit all populations equitably [48, 67].

Furthermore, increasing the diversity of genomic data not only mitigates disparities but also significantly enhances the fine-mapping of GWAS signals and the identification of target genes [67]. This is crucial for uncovering the genetic mechanisms influencing the development of NCDs and other complex conditions. Underrepresented groups, such as those of African and South Asian ancestries, exhibit higher genetic diversity, which translates into substantial gains in genomic research [205, 206]. Studies incorporating these populations have unearthed population-enriched clinically important variants that were previously undiscovered in predominantly European datasets. For example, research into African genetic ancestry has led to critical insights, including the link between *APOL1* variants and chronic kidney disease [207], the identification of *G6PD* variants that refine diabetes diagnostics [208], and loss of function variants in PCSK9 that contribute to lower low-density lipoprotein cholesterol levels—this latter discovery has spurred the development of PCSK9 inhibitor drugs [209]. These findings underscore the value of including diverse genetic backgrounds in research to achieve a comprehensive understanding of genetic factors across all populations, enhancing the overall impact of genomic studies on global health.

The lack of genetic diversity is a pervasive issue across various omics datasets, not just genomics [35, 91, 142, 159]. For instance, bulk and single-cell transcriptomic analyses are beginning to uncover significant heterogeneity in gene expression across different cell types and even within the same type. This diversity is especially pronounced across different genetic ancestries, shaped by distinct environmental and genetic interactions. Major research efforts, such as single-cell consortia including KPMP, LungMAP, HTCA, GTEx [88], HuBMAP, Azimuth, HCA, and the Allen Brain Atlas, have predominantly focused on populations of European genetic ancestry, resulting in the underrepresentation of other groups. For example, of the 4,723 samples analyzed across these consortia, the majority are from individuals of European descent, starkly contrasted with the minimal representation from African, Hispanic, and East Asian ancestries. Addressing this imbalance is critical for enhancing our understanding of context-specific cellular mechanisms and improving the detection and treatment of diseases that vary regionally due to factors like genetic drift and migration. This understanding is particularly vital in pharmacogenomics, where knowing context-specific gene regulation can significantly advance personalized medicine. In a significant step toward addressing this imbalance, the Chan Zuckerberg Initiative has recently funded the Ancestry Networks for the Human Cell Atlas (HCA) with a $28 million grant, supporting the inclusion of ancestrally diverse tissue samples to ensure a broader representation and deeper insights into the genetic underpinnings of health and disease across populations.

Similarly, the representation of genetic diversity in epigenomic data is markedly limited [210], as demonstrated by a study by Breeze et al. (2022). This study revealed that among the 5,048 epigenetic experiments from the US-based ENCODE data and the International Human Epigenome Consortium (IHEC), 87.1% (n = 4,397) predominantly featured samples of European genetic ancestry, with other ancestries severely underrepresented. Such disparities underscore a significant bias in the samples analyzed, with only a fraction representing African, Asian, and other ancestries. This lack of diversity impedes our ability to fully understand and interpret disease-associated genomic regions across populations. Epigenomic markers such as promoters, enhancers, and repressors are crucial for annotating non-coding regions identified by GWAS, which often have unclear functional implications. Broadening the scope of epigenomic data to include diverse populations could enhance the interpretation of GWAS loci, offering vital insights into the regulatory mechanisms affecting diseases that disproportionately impact non-European populations, like prostate cancer, hypertension, and chronic kidney disease.

#### Measuring exposomes

Measuring exposomes in multi-omics research on NCDs involves significant challenges due to the complexity and diversity of environmental exposures. Exposomes encompass a range of external factors, like pollution and radiation, alongside internal factors, such as microbiome interactions and metabolic processes. Technologies like mass spectrometry (MS) and geographic information systems (GIS) are essential for quantifying these exposures. MS, particularly untargeted MS, excels in detecting a broad spectrum of small molecules in biological samples, providing a comprehensive snapshot of chemical exposures. However, the vast amount of data generated requires advanced bioinformatics for accurate analysis, and detection sensitivity varies significantly among different chemical classes.

GIS tools assess environmental exposure by integrating diverse data sources to model spatial and temporal distribution patterns of factors like air and water quality. This modeling is crucial for evaluating health risks linked to environmental factors. Additionally, wearable sensor technologies revolutionize exposure monitoring by providing real-time, individual exposure data to elements such as air quality and UV radiation, offering granular insights into daily exposure patterns. Despite these advancements, the dynamic nature of environmental exposures and the heterogeneity in measurement techniques pose substantial challenges. These include the need for standardized data collection methods and the development of structured data sharing protocols to facilitate comparisons and enhance the accuracy of exposome research in understanding NCDs.

#### Establishing and maintaining biobanks

Establishing and maintaining biobanks is a critical yet challenging endeavor in omics and multi-omics research, particularly in low and middle-income countries (LMICs) [45, 47, 66, 69]. While most biobanks are found in high-income countries, equipped with advanced infrastructure and technical capacity, LMICs face substantial barriers such as inadequate funding, limited institutional capacity, and a shortage of skilled professionals. This disparity is especially pronounced in Africa and South Asia, which are severely underrepresented in genomic research [48, 66]. Most genomic studies in LMICs rely on funding from high-income countries through collaborative efforts, often resulting in research agendas set by external priorities rather than local needs [66].

Significant and sustained investment in biobanking infrastructure in under-represented regions is crucial to address the lack of diversity in omics research. Initiatives like the China Kadoorie Biobank and the South African Human Genome Project provide hopeful examples of how national governments are recognizing the value of omics studies [66]. In addition, further improvement in this field could be achieved by global consortia directing technical and financial resources to build local biobanking capacities in LMICs. This approach not only helps in establishing the necessary infrastructure for sample processing, genotyping, sequencing, and computational analysis, but also facilitates equitable data, ultimately benefiting the global scientific community. For instance, the Africa Wits-INDEPTH Partnership for Genomic Research (AWI-Gen) exemplifies a strategic regional collaboration funded by the NIH [66]. This project has established a cross-sectional population cohort of about 12,000 adults across four African countries, leveraging existing Health and Demographic Surveillance System centers and community engagement to span a wide representation of social and genetic variability [67, 211]. Similarly, initiatives such as the H3Africa, H3Africa Bioinformatics Network(H3AfricaBioNet), and the Data Science for Health Discovery and Innovation in Africa, strategic funding commitments by the NIH, exemplify efforts to bolster genetic research capacity in Africa [212, 213]. However, it is important to note that future funding commitments in genomics will benefit from expansion to broader continental regions to address health problems and capacity-building needs of countries with no pre-existing omics research infrastructure.

Another significant hurdle is the lack of expertise for addressing the ethical, legal, and social implications (ELSIs) of multi-omics research, which hinders the conduct of research and efficient sharing of data [47]. To address this, it is essential to create national and local opportunities for advanced training, foster continuous professional development, and develop comprehensive ELSI guidelines that can be integrated into study designs. These measures will ensure that multi-omics research is conducted responsibly and its benefits are equitably shared, maintaining the integrity and relevance of the research. Additionally, promoting workforce diversity in omics research is crucial for building trust and fostering engagement among underrepresented groups. Diverse research teams are more likely to focus on health issues pertinent to their communities, which in turn encourages broader participation and consent in biobank studies. This not only strengthens the relationship between researchers and participants but also enhances the quality of research data, making genomic studies more impactful and relevant across populations [67].

#### Multi-omics data and integration methods

Integrating multi-omics data to unravel complex GxE interactions in NCDs is complicated by diverse data formats and significant preprocessing requirements [35, 142, 214, 215]. The lack of standardized methods for preprocessing and integrating data from various omics platforms often compromises the effectiveness of analyses [41, 216]. Additionally, the integration process is challenged by the "curse of dimensionality." This term describes issues that arise in high-dimensional datasets, where the volume of variables far exceeds the number of samples, leading to data sparsity and inconsistency across samples [217]. This makes it difficult to draw reliable conclusions from the data, emphasizing the need for robust analytical tools and methods that can handle and integrate vast and varied omics data effectively.

On the other hand, tissue and cell-type heterogeneity present another significant challenge in multi-omics integration, particularly relevant to studying complex diseases [218]. Different cell types within a single tissue sample can exhibit unique omics profiles, influenced by the tissue's specific section or the physiological condition of an individual [218]. These variations can skew biomarker levels and lead to misleading associations that reflect cellular differences rather than the disease itself. Although statistical methods have been developed to adjust for cell-type heterogeneity, they may not fully account for the true biological variations or might even over-correct them. Ideally, single-cell omics would provide a clearer picture by isolating the profiles of each cell type, but this approach is often impractical due to high costs and material requirements [37, 178]. The challenges of sample heterogeneity and technical artifacts, such as batch effects during sequencing, underscore the complexity of data preprocessing in multi-omics studies. Ensuring consistent data processing and leveraging appropriate statistical controls are crucial for mitigating these issues and enhancing the reliability of multi-omics analyses.

Furthermore, while NGS technologies have made sequencing faster and more affordable, they have also introduced challenges such as increased costs for participant recruitment and sample processing, and complexities in data management and storage [178, 218]. Privacy concerns frequently limit data sharing between institutions, sometimes leading to the withdrawal of large datasets from public access due to potential identification risks [215, 218]. Moreover, proprietary standards for biomedical devices and health IT systems hinder seamless data integration across different sources [35]. Addressing these issues requires comprehensive efforts to harmonize data across various healthcare providers and omics modalities, necessitating a collaborative approach from all stakeholders in healthcare and research to enhance real-world evidence-based practices and improve healthcare outcomes.

Another multi-omics integration challenge, particularly when applying enrichment-based methods to uncover gene-environment interactions in NCDs, is the potential bias introduced by linkage disequilibrium, colocalization of multiple functional variants, and unaccounted confounders [219]. Fine-mapping and imputation-based methods, which are crucial for developing biomarkers and understanding molecular mechanisms, depend heavily on the accuracy of population-specific linkage disequilibrium matrices [220]. These methods also rely on robust genetic reference models for molecular features such as gene expression or methylation, which are difficult to obtain for features other than gene expression [220]. The variability of QTL architecture across different tissues further complicates these analyses, necessitating careful consideration of tissue relevance to the disease mechanisms under study [220]. Researchers must ensure they are well-versed in the biological assumptions, statistical constraints, and computational demands of the integration tools they choose to employ to enhance the reliability and applicability of their findings in NCD research.

#### Validation of GxE interactions and translational applications

Validating GxE interactions identified in human research and translating them into actionable insights remains a critical challenge. Translational studies using model organisms bridge observational findings with mechanistic understanding, allowing researchers to explore how genetic and environmental factors interplay in the development of NCDs [133]. Model organisms such as mice [221], rats [222], Drosophila melanogaster [223], and Caenorhabditis elegans [224] offer controlled environments where genetic and environmental variables can be precisely manipulated. This control facilitates the dissection of complex biological processes that are challenging to study directly in humans due to ethical and practical constraints. Moreover, hypotheses generated from these studies can be tested using human genetic data, improving detection power and enabling a more detailed analysis of subpopulations to understand GxE interactions better. Incorporating functional annotations from resources such as ENCODE, GTEx, and Roadmap Epigenomics further enhances this process by prioritizing candidate variants and regulatory regions for GxE studies, particularly those in non-coding regions often affected by environmental exposures [225].

For example, genetically diverse rodent models like the Collaborative Cross [226] and Diversity Outbred lines [227], which have high sequence homology with humans, have been instrumental in identifying QTLs and candidate genes involved in GxE interactions relevant to human NCDs. A notable case involves mutations in the tumor suppressor gene *BAP1*, which have been linked with increased susceptibility to mesothelioma following asbestos exposure [228]. Exploring how *BAP1* mutations interact with asbestos exposure could elucidate key molecular pathways in carcinogenesis, with the potential to inform targeted screening, prevention strategies, and therapies tailored to the underlying mechanisms. Similarly, studies in Drosophila and C. elegans have facilitated high-throughput screening of genetic variants and environmental exposures, uncovering genetic pathways that modulate responses to environmental stressors and offering translational insights about human health [229, 230].

However, the translation of findings from model systems to human populations is not without challenges. While model organisms provide controlled environments, they cannot fully replicate the genetic complexity, environmental diversity, or numerous confounding factors that influence human health. For instance, gene synteny between humans and model organisms often diverges, particularly for non-coding and regulatory regions, limiting the applicability of some findings. Studies such as Seok et al. (2013) have demonstrated that genomic responses in mouse models often poorly mimic human inflammatory diseases, reflecting the inherent differences in gene regulatory networks and physiological responses. Furthermore, humans are exposed to a far more diverse range of environmental factors—such as diet, pollution, and stress—than those typically replicated in model organism studies, which limits the generalizability of findings [231, 232] (Table 1).

**Table 1:** Summary of Key Challenges in Omics and Multi-Omics Research, Implications, and Potential Solutions

Area	Challenges	Implications	Potential Solutions
Diversity of Omics/Multi-Omics Datasets	Under-representation of non-European genetic ancestry populations in genomic and other omics datasets	Reduced predictive accuracy of genetic risk predictors and limited generalizability across diverse populations, potentially exacerbating health disparities Impeded discovery of population-specific variants and context-specific disease mechanisms	Expand targeted research participant recruitment initiatives to diversify genomics and other omics datasets Increase funding and incentives for ancestrally inclusive research Enhance accessibility to repositories of underrepresented populations
Measuring Exposomes	Limitations in analytical tools quantifying complex and dynamic environmental exposures Complexity of integrating data from diverse exposure measures due to inadequate standardization, particularly from real-time monitoring technologies	Variablity in data quality hinders accurate exposome measurements and comparisons Limited applicability of exposome data in identifying exposure-disease linkages	Standardize data collection and harmonize protocols for reproducibility Develop advanced real-time monitoring techniques and integration methods Foster open data-sharing and interoperability across systems to enhance exposome research
Multi-Omics Integration	Limited standardization in pre-processing and integration across omics platforms Challenges due to high dimentionality and sparse datasets (“curse of dimentionality”) Tissue and cell-type heterogeneity skews biomarker discovery Privacy concerns and proprietary standards limit data sharing and interoperability	Poor integration reduces reliability and generalizability of findings, hindering insights into disease mechanisms Misleading results due to cellular variation rather than disease-pecific mechanisms Fragmented datasets impede comprehensive analyses and real-world applications of findings	Promote standardized protocols and robust analytical tools to manage and integrate high-dimentional data Expand single-cell omics datasets and employ appropriate approaches to correct for cell-type heterogeneity Harmonize data-sharing policies and adopt open standards for interoperability between biomedical systems and health IT platforms
Validation and Translational application of GxE findings	Limited experimental validation of GxE findings in model organisms and translational settings Pratical constraints in applying GxE insights to precision medicine and public health interventions	Reduced confidence in the biological mechanisms underlying GxE interactions, limiting practical applications Slower progress in developing tailored therapies and prevention strategies	Expand use of in vitro systems and model organisms to understand functional mechanisms of findings, Integrate translational studies with human data to enhance detection and understanding of GxE interactions Expand investment in precision health and pharmacogenomics research, leveraging validated GxE insights

Functional annotations and perturbation studies, conducted in both in vitro and in vivo settings, hold promise for unraveling the complexities of GxE interactions in NCDs and other complex diseases [233]. Functional annotations derived from large-scale projects, such as ENCODE and GTEx, systematically map regulatory elements and link genetic variants to potential functional effects, guiding the identification of candidate variants and regulatory regions [88, 234, 235]. Perturbation studies, including CRISPR-Cas9-based approaches, enable direct testing of causal hypotheses [236]. For example, CRISPR interference (CRISPRi) and activation (CRISPRa) screens in human induced pluripotent stem cell (hiPSC)-derived neurons have identified essential genes for neuronal survival under chronic oxidative stress–a key environmental factor relevant to neurodegenerative diseases–revealing critical mediators like *GPX4* and other selenoprotein synthesis genes [237].

Translational applications of GxE analysis have direct implications for personalized medicine and public health interventions [238]. In precision environmental health, identifying how specific genetic variations influence susceptibility to environmental exposures enables the development of tailored interventions. For instance, genetic variation in *CYP2D6* may influence susceptibility to Parkinson’s disease through pesticide exposure, with poor metabolizers potentially at greater risk [239]. These findings could inform strategies to reduce exposure in vulnerable populations, though further research is needed for confirmation. The ALDH2*2 variant, common in certain populations, impairs acetaldehyde metabolism and may increase the risk of esophageal cancer with alcohol intake, suggesting the potential for personalized dietary recommendations and targeted prevention strategies in affected populations [240].

In pharmacogenomics, GxE interactions can guide personalized medication regimens to optimize efficacy and minimize adverse effects. Personalized warfarin dosing based on variations in genes like *VKOR1* and *CYP2C9* has been shown to improve therapeutic outcomes and reduce the risk of bleeding complications [241]. Variants in the *TPMT* gene necessitate dose adjustments of thiopurine drugs to prevent toxicity in treating conditions like leukemia and autoimmune diseases [242]. *CYP2D6* gene variants inform the selection and dosing of antidepressants, enhancing treatment response and reducing side effects [242]. In oncology, identifying *BRCA1/2* mutations allows for the use of Poly(ADP-ribose) polymerase (PARP) inhibitors in targeted cancer therapy, while *HER2* expression guides the use of trastuzumab in breast cancer treatment, exemplifying how GxE insights contribute to precision medicine [243]. Approaches that integrate biological pathways and regulatory annotations can further enhance the discovery and application of such GxE findings.

These translational applications underscore the importance of validating GxE interactions through model organisms and advanced experimental systems. However, significant challenges persist. Limited experimental validation of GxE findings in model organisms and translational settings undermines confidence in the biological mechanisms underlying these interactions, slowing their application to precision medicine and public health interventions [244]. High costs and the technical complexity of integrating environmental monitoring data with omics insights further impede progress. Additionally, the lack of standardized protocols for validating GxE findings, combined with the scarcity of diverse model systems, restricts the development of tailored therapies and prevention strategies [233, 244]. These issues collectively limit the potential of GxE research to address global health disparities effectively, particularly in low-resource settings where both environmental and omics data are underrepresented.

## Summary and concluding remarks

This scoping review highlighted that NCDs, such as cardiovascular diseases, cancers, chronic respiratory diseases, and diabetes, result from the complex interaction of gene and environmental factors, such as diet, physical inactivity, and tobacco use. To unravel the complexity of GxE interactions and gain an understanding of the multiple factors underlying many NCDs, a multi-omics approach is indeed essential. By employing multi-omics and data integration techniques, it can be possible to fully understand how the interaction of genetic and environmental factors influences NCD development, progression, and treatment response. This involves exploring a range of omics disciplines—genomics, transcriptomics, epigenomics, proteomics, and exposomics and understanding how they individually and collectively influence the risk to NCDs.

Despite the transformative potential of global multi-omics research in advancing precision medicine, there are significant challenges and opportunities related to its practical translational applications. Our review highlighted that the genome can not be viewed as a static entity, as both genetic and environmental factors dynamically influence disease onset and progression. We provide several examples of how different modalities complement genomic data by revealing dynamic changes in gene expression, pathways, and networks due to environmental exposures. Thus, comprehensive omics integration is essential for identifying novel biomarkers and therapeutic targets and enhancing diagnostic, prognostic, and treatment strategies. Integrative analyses ideally would involve multiple omics data from the same individuals, although practical challenges such as cost and tissue accessibility may often limit this ideal. International consortia and national biobanks have been established to address these challenges, collecting detailed phenotypic and, increasingly, omics biomarker data to fill major gaps in NCD research. Moreover, we underscored a pervasive issue of limited diversity across omics and multi-omics datasets, affecting the transferability of research findings and tools across different genetic ancestries. Currently, most genomic and omics studies predominantly feature individuals of European descent, significantly underrepresenting African, Latin American, and Indigenous populations. This underrepresentation compromises the predictive accuracy of polygenic scores and other genomic tools when applied to non-European groups. Furthermore, including diverse genetic ancestries is crucial not only for enhancing the precision of GWAS signal mapping but also for discovering clinically relevant genetic variants that remain unidentified in predominantly European datasets. For example, studies involving African ancestry have led to key discoveries in chronic kidney disease and diabetes management. Addressing this lack of diversity is essential not only to improve the scientific robustness of omics research but also to mitigate health disparities, ensuring that the benefits of genomic advances reach all populations equitably.

To address fairness in multi-omics for equitable health advancements, concerted efforts should focus on increasing the diversity of both omics and multi-omics datasets. Future research should also aim to develop equity-centered genomics medicine advanced computational methods and tools that efficiently integrate various omics datasets. These tools must generate biomarkers or risk predictors that are broadly transferable across genetic ancestries, with a particular emphasis on accounting for known confounders such as gene-environment correlations, including population stratification and assortative mating. By doing so, we can improve the scientific robustness of omics research and ensure that the benefits of genomic advances reach all populations equitably, thereby helping to mitigate health disparities globally.

Lastly, our exploration of multi-omics integration methods has illuminated the intricate challenges of combining diverse datasets to decode complex GxE interactions in NCDs. The heterogeneity of tissue and cell types significantly compounds these challenges, with variations in omics profiles within a single tissue potentially misleading biomarker identification. Statistical adjustments for cell-type heterogeneity aim to correct these variations, yet there is a risk of over-correction, obscuring genuine biological differences. High costs and logistical constraints often thwart the ideal solution of employing single-cell omics to circumvent these issues. Moreover, while advancements like NGS have reduced costs and increased the speed of data acquisition, they introduce new difficulties in data management, participant recruitment, and inter-institutional data sharing due to privacy concerns. This requires a coordinated effort to harmonize multi-omics data across different healthcare settings, requiring a collaborative approach among all stakeholders to leverage real-world evidence for improving health outcomes effectively.

## Supplementary Information

**Additional file 1**.

**Additional file 2**.

**Additional file 3**.

## Data Availability

All data analyzed in this study are publicly available from the cited sources; no new data were generated. Citation trends for multi-omics, personalized/precision medicine, and gene-environment interactions from 2000 to 2023 were obtained from PubMed using a Python script that utilizes the requests and BeautifulSoup libraries. Sequencing cost data were sourced from the National Human Genome Research Institute’s Genome Sequencing Program (www.genome.gov/sequencingcostsdata, accessed June 17, 2024). The geographic distribution data of cumulative participants in genome-wide association studies (GWAS) were obtained from Mills and Rahal (2020) as maintained by the Leverhulme Centre for Demographic Science. The python scripts used to generate the figures are publicly available on GitHub at https://github.com/robelalemu01/multi-omics-gxe-ncd-review.

## References

[1] Ferrari AJ, et al. Global incidence, prevalence, years lived with disability (YLDs), disability-adjusted life-years (DALYs), and healthy life expectancy (HALE) for 371 diseases and injuries in 204 countries and territories and 811 subnational locations, 1990–2021: a systematic analysis for the Global Burden of Disease Study 2021. The Lancet. 2024;403:2133–61. 10.1016/S0140-6736(24)00757-8 PMC11122111 38642570

[2] World Health Organization. *Global Action Plan for the Prevention and Control of Noncommunicable Diseases, 2013–2020.*

[3] World Economic Forum, H. S. of P. H. *The Global Economic Burden of Non-Communicable Diseases*. www.weforum.org/EconomicsOfNCD (2011).

[4] Forouzanfar MH, et al. Global, regional, and national comparative risk assessment of 79 behavioural, environmental and occupational, and metabolic risks or clusters of risks, 1990–2015: a systematic analysis for the Global Burden of Disease Study 2015. The Lancet. 2016;388:1659–724. 10.1016/S0140-6736(16)31679-8 PMC5388856 27733284

[5] Ndubuisi NE. Noncommunicable diseases prevention in low- and middle-income countries: an overview of health in all policies (HiAP). Inquiry (United States). 2021;58:536. 10.1177/0046958020927885 PMC8385577 34420412

[6] Budreviciute A, et al. Management and prevention strategies for non-communicable diseases (NCDs) and their risk factors. Front Public Health. 2020;8:21. 33324597 10.3389/fpubh.2020.574111 PMC7726193

[7] Calcaterra V, Zuccotti G. Non-communicable diseases and rare diseases: a current and future public health challenge within pediatrics. Children. 2022;9:245. 36291427 10.3390/children9101491 PMC9600389

[8] Tohi M, Bay JL, Tuakoi S, Vickers MH. The developmental origins of health and disease: adolescence as a critical lifecourse period to break the transgenerational cycle of NCDs—a narrative review. Int J Environ Res Public Health. 2022;19:52. 10.3390/ijerph19106024 PMC9141771 35627561

[9] Calcaterra V, Zuccotti G. Non-communicable diseases and rare diseases: a current and future public health challenge within pediatrics. Children. 2022;9:21. 10.3390/children9101491 PMC9600389 36291427

[10] World Health Organization (WHO). *GLOBAL STATUS REPORT on Noncommunicable Diseases 201 4 ‘Attaining the Nine Global Noncommunicable Diseases Targets; a Shared Responsibility’*. (2014).

[11] Drobni ZD, et al. Heritability of coronary artery disease: insights from a classical twin study. Circ Cardiovasc Imaging. 2022;15:133–41. 10.1161/CIRCIMAGING.121.013348 PMC8925867 35290075

[12] Bai D, et al. Association of genetic and environmental factors with Autism in a 5-country cohort. JAMA Psychiat. 2019;76:1035–43. 10.1001/jamapsychiatry.2019.1411 PMC6646998 31314057

[13] Khera AV, Kathiresan S. Genetics of coronary artery disease: discovery, biology and clinical translation. Nat Rev Genet. 2017;18:331–44. 28286336 10.1038/nrg.2016.160 PMC5935119

[14] Balmain A, Gray J, Ponder B. The genetics and genomics of cancer. Nat Genet. 2003;33:238–44. 12610533 10.1038/ng1107

[15] Grotzinger AD, et al. Genetic architecture of 11 major psychiatric disorders at biobehavioral, functional genomic and molecular genetic levels of analysis. Nat Genet. 2022;54:548–59. 35513722 10.1038/s41588-022-01057-4 PMC9117465

[16] Tcheandjieu C, et al. Large-scale genome-wide association study of coronary artery disease in genetically diverse populations. Nat Med. 2022;28:1679–92. 35915156 10.1038/s41591-022-01891-3 PMC9419655

[17] Khan A, et al. Genome-wide polygenic score to predict chronic kidney disease across ancestries. Nat Med. 2022;28:1412–20. 35710995 10.1038/s41591-022-01869-1 PMC9329233

[18] Tsuo K, et al. Multi-ancestry meta-analysis of asthma identifies novel associations and highlights the value of increased power and diversity. Cell Genom. 2022;2:25. 10.1016/j.xgen.2022.100212 PMC9903683 36778051

[19] Sadee W, et al. Missing heritability of common diseases and treatments outside the protein-coding exome. Hum Genet. 2014;133:1199–215. 25107510 10.1007/s00439-014-1476-7 PMC4169001

[20] Génin E. Missing heritability of complex diseases: case solved? Hum Genet. 2020;139:103–13. 31165258 10.1007/s00439-019-02034-4

[21] Manolio TA, et al. Finding the missing heritability of complex diseases. Nature. 2009;461:747–53. 19812666 10.1038/nature08494 PMC2831613

[22] Blanco-Gómez A, et al. Missing heritability of complex diseases: enlightenment by genetic variants from intermediate phenotypes. BioEssays. 2016;38:664–73. 27241833 10.1002/bies.201600084 PMC5064854

[23] Zuk O, Hechter E, Sunyaev SR, Lander ES. The mystery of missing heritability: genetic interactions create phantom heritability. Proc Natl Acad Sci U S A. 2012;109:1193–8. 22223662 10.1073/pnas.1119675109 PMC3268279

[24] Cano-Gamez E, Trynka G. From GWAS to function: using functional genomics to identify the mechanisms underlying complex diseases. Front Genet. 2020;11:254. 32477401 10.3389/fgene.2020.00424 PMC7237642

[25] Spain SL, Barrett JC. Strategies for fine-mapping complex traits. Hum Mol Genet. 2015;24:111–9. 10.1093/hmg/ddv260 PMC4572002 26157023

[26] Hofker MH, Fu J, Wijmenga C. The genome revolution and its role in understanding complex diseases. Biochimica et Biophysica Acta Mol Basis Dis. 2014;1842:1889–95. 10.1016/j.bbadis.2014.05.002 24834846

[27] Kierczak M, et al. Contribution of rare whole-genome sequencing variants to plasma protein levels and the missing heritability. Nat Commun. 2022;13:17. 35534486 10.1038/s41467-022-30208-8 PMC9085767

[28] Virolainen SJ, VonHandorf A, Viel KCMF, Weirauch MT, Kottyan LC. Gene–environment interactions and their impact on human health. Genes Immunity. 2023;1:1–11. 10.1038/s41435-022-00192-6 PMC9801363 36585519

[29] Jung HU, et al. Gene-environment interaction explains a part of missing heritability in human body mass index. Commun Biol. 2023;6:18. 36966243 10.1038/s42003-023-04679-4 PMC10039928

[30] Wang K, et al. Interpretation of association signals and identification of causal variants from genome-wide association studies. Am J Hum Genet. 2010;86:730–42. 20434130 10.1016/j.ajhg.2010.04.003 PMC2869011

[31] Du Y, Fan K, Lu X, Wu C. Integrating multi-omics data for gene-environment interactions. BioTech. 2021;10:26. 35822775 10.3390/biotech10010003 PMC9245467

[32] Noble AJ, et al. A final frontier in environment-genome interactions? integrated, multi-omic approaches to predictions of non-communicable disease risk. Front Genet. 2022;13:214. 10.3389/fgene.2022.831866 PMC8861380 35211161

[33] Raufaste-Cazavieille V, Santiago R, Droit A. Multi-omics analysis: Paving the path toward achieving precision medicine in cancer treatment and immuno-oncology. Front Mol Biosci. 2022;9:43. 10.3389/fmolb.2022.962743 PMC9595279 36304921

[34] Kamali Z, et al. Large-scale multi-omics studies provide new insights into blood pressure regulation. Int J Mol Sci. 2022;23:14. 10.3390/ijms23147557 PMC9323755 35886906

[35] Sathyanarayanan A, et al. Multi-omics data integration methods and their applications in psychiatric disorders. Eur Neuropsychopharmacol. 2023;69:26–46. 36706689 10.1016/j.euroneuro.2023.01.001

[36] Wu H, Eckhardt CM, Baccarelli AA. Molecular mechanisms of environmental exposures and human disease. Nat Rev Genet. 2023;24:332–44. 36717624 10.1038/s41576-022-00569-3 PMC10562207

[37] Tong L, et al. Integrating multi-omics data with ehr for precision medicine using advanced artificial intelligence. IEEE Rev Biomed Eng. 2024;17:80–97. 37824325 10.1109/RBME.2023.3324264

[38] Nicora G, Vitali F, Dagliati A, Geifman N, Bellazzi R. Integrated multi-omics analyses in oncology: a review of machine learning methods and tools. Front Oncol. 2020;10:56. 32695678 10.3389/fonc.2020.01030 PMC7338582

[39] Subramanian I, Verma S, Kumar S, Jere A, Anamika K. Multi-omics data integration, interpretation, and its application. Bioinf Biol Insights. 2020;14:658. 10.1177/1177932219899051 PMC7003173 32076369

[40] Zhan C, et al. From multi-omics approaches to personalized medicine in myocardial infarction. Front Cardiovasc Med. 2023;10:356. 10.3389/fcvm.2023.1250340 PMC10642346 37965091

[41] Reel PS, Reel S, Pearson E, Trucco E, Jefferson E. Using machine learning approaches for multi-omics data analysis: a review. Biotechnol Adv. 2021;49:563. 10.1016/j.biotechadv.2021.107739 33794304

[42] Prosperi M, Min JS, Bian J, Modave F. Big data hurdles in precision medicine and precision public health. BMC Med Inform Decis Mak. 2018;18:21457. 10.1186/s12911-018-0719-2 PMC6311005 30594159

[43] Santiago-Rodriguez TM, Hollister EB. Multi ‘omic data integration: a review of concepts, considerations, and approaches. Semin Perinatol. 2021;45:210. 10.1016/j.semperi.2021.151456 34256961

[44] Picard M, Scott-Boyer MP, Bodein A, Périn O, Droit A. Integration strategies of multi-omics data for machine learning analysis. Comput Struct Biotechnol J. 2021;19:3735–46. 34285775 10.1016/j.csbj.2021.06.030 PMC8258788

[45] Yang G, Mishra M, Perera MA. Multi-omics studies in historically excluded populations: the road to equity. Clini Pharmacol Therap. 2023;113:541–56. 10.1002/cpt.2818 PMC10323857 36495075

[46] Green S, Prainsack B, Sabatello M. Precision medicine and the problem of structural injustice. Med Health Care Philos. 2023;26:433–50. 37231234 10.1007/s11019-023-10158-8 PMC10212228

[47] Edwards, T. L., Breeyear, J., Piekos, J. A., & Velez Edwards, D. R. Consideration of Race and Ethnicity in Precision Medicine. *Trends Genet* 36: 807–809 (2020). 10.1016/j.tig.2020.07.001 PMC7373675 32709459

[48] Martin AR, et al. Clinical use of current polygenic risk scores may exacerbate health disparities. Nat Genet. 2019;51:584–91. 30926966 10.1038/s41588-019-0379-x PMC6563838

[49] Ding Y, et al. Polygenic scoring accuracy varies across the genetic ancestry continuum. Nature. 2023;618:774–81. 37198491 10.1038/s41586-023-06079-4 PMC10284707

[50] Wekesa JS, Kimwele M. A review of multi-omics data integration through deep learning approaches for disease diagnosis, prognosis, and treatment. Front Geneti. 2023;14:52. 10.3389/fgene.2023.1199087 PMC10398577 37547471

[51] Barouki, R., Gluckman, P. D., Grandjean, P., Hanson, M. & Heindel, J. J. *Developmental Origins of Non-Communicable Disease: Implications for Research and Public Health*. http://www.ehjournal.net/content/11/1/42 (2012). 10.1186/1476-069X-11-42 PMC3384466 22715989

[52] Ngo KJ, et al. Lysosomal genes contribute to Parkinson’s disease near agriculture with high intensity pesticide use. NPJ Parkinsons Dis. 2024;10:501. 10.1038/s41531-024-00703-4 PMC11045791 38664407

[53] Schaffner SL, et al. Genetic variation and pesticide exposure influence blood DNA methylation signatures in females with early-stage Parkinson’s disease. NPJ Parkinsons Dis. 2024;10:654. 10.1038/s41531-024-00704-3 PMC11076573 38714693

[54] Young AI, Wauthier F, Donnelly P. Multiple novel gene-by-environment interactions modify the effect of FTO variants on body mass index. Nat Commun. 2016;7:21. 10.1038/ncomms12724 PMC5025863 27596730

[55] Qi T, Song L, Guo Y, Chen C, Yang J. From genetic associations to genes: methods, applications, and challenges. Trends Genet. 2024. 10.1016/j.tig.2024.04.008. 38734482 10.1016/j.tig.2024.04.008

[56] Kamali Z, et al. Large-scale multi-omics studies provide new insights into blood pressure regulation. Int J Mol Sci. 2022;23:85. 10.3390/ijms23147557 PMC9323755 35886906

[57] Visscher PM, et al. 10 years of GWAS discovery: biology, function, and translation. Am J Hum Genet. 2017;101:5–22. 28686856 10.1016/j.ajhg.2017.06.005 PMC5501872

[58] Abdellaoui A, Yengo L, Verweij KJH, Visscher PM. 15 years of GWAS discovery: realizing the promise. Am J Hum Genet. 2023;110:179–94. 36634672 10.1016/j.ajhg.2022.12.011 PMC9943775

[59] Loos RJF. 15 years of genome-wide association studies and no signs of slowing down. Nat Commun. 2020;11:89. 33214558 10.1038/s41467-020-19653-5 PMC7677394

[60] Satam H, et al. Next-generation sequencing technology: current trends and advancements. Biology. 2023;12:856. 37508427 10.3390/biology12070997 PMC10376292

[61] Wang K, et al. Diverse Genome-wide Association Studies Associate the IL12/IL23 Pathway with Crohn Disease. Am J Hum Genet. 2009;84:399–405. 19249008 10.1016/j.ajhg.2009.01.026 PMC2668006

[62] Lennon NJ, et al. Selection, optimization and validation of ten chronic disease polygenic risk scores for clinical implementation in diverse US populations. Nat Med. 2024;30:480–7. 38374346 10.1038/s41591-024-02796-z PMC10878968

[63] Amare AT, et al. Association of polygenic score for major depression with response to lithium in patients with bipolar disorder. Mol Psychiatry. 2021;26:2457–70. 32203155 10.1038/s41380-020-0689-5

[64] Schubert KO, et al. Combining schizophrenia and depression polygenic risk scores improves the genetic prediction of lithium response in bipolar disorder patients. Transl Psychiatry. 2021;11:74. 34845190 10.1038/s41398-021-01702-2 PMC8630000

[65] Amare AT, Schubert KO, Baune BT. Pharmacogenomics in the treatment of mood disorders: strategies and opportunities for personalized psychiatry. EPMA J. 2017;8:211–27. 29021832 10.1007/s13167-017-0112-8 PMC5607053

[66] Fatumo S, et al. A roadmap to increase diversity in genomic studies. Nat Med. 2022;28:243–50. 35145307 10.1038/s41591-021-01672-4 PMC7614889

[67] Fatumo S, et al. A roadmap to increase diversity in genomic studies. Nat Med. 2022;28:243–50. 35145307 10.1038/s41591-021-01672-4 PMC7614889

[68] Geneviève LD, Martani A, Shaw D, Elger BS, Wangmo T. Structural racism in precision medicine: Leaving no one behind. BMC Med Ethics. 2020;21:89. 32075640 10.1186/s12910-020-0457-8 PMC7031946

[69] Khoury MJ, et al. Health equity in the implementation of genomics and precision medicine: a public health imperative. Genet Med. 2022;24:1630–9. 35482015 10.1016/j.gim.2022.04.009 PMC9378460

[70] Tishkoff SA, Williams SM. Genetic analysis of African populations: human evolution and complex disease. Nat Rev Genet. 2002;3:611–21. 12154384 10.1038/nrg865

[71] Reed FA, Tishkoff SA. African human diversity, origins and migrations. Curr Opini Genet Dev. 2006;16:597–605. 10.1016/j.gde.2006.10.008 17056248

[72] Campbell MC, Tishkoff SA. African genetic diversity: Implications for human demographic history, modern human origins, and complex disease mapping. Annu Rev Genom Hum Genet. 2008;9:403–33. 10.1146/annurev.genom.9.081307.164258 PMC2953791 18593304

[73] Gurdasani D, et al. The African Genome Variation Project shapes medical genetics in Africa. Nature. 2015;517:327–32. 25470054 10.1038/nature13997 PMC4297536

[74] Sirugo G, et al. Genetic studies of African populations: an overview on disease susceptibility and response to vaccines and therapeutics. Hum Genet. 2008;123:557–98. 18512079 10.1007/s00439-008-0511-y

[75] Genomic data in the All of Us Research Program. *Nature* **627**, 340–346 (2024). 10.1038/s41586-023-06957-x PMC10937371 38374255

[76] Domingue BW, Fletcher J, Conley D, Boardman JD. Genetic and educational assortative mating among US adults. Proc Natl Acad Sci U S A. 2014;111:7996–8000. 24843128 10.1073/pnas.1321426111 PMC4050565

[77] Veller C, Coop GM. Interpreting population- and family-based genome-wide association studies in the presence of confounding. PLoS Biol. 2024;22:86. 10.1371/journal.pbio.3002511 PMC11008796 38603516

[78] Howe LJ, et al. Within-sibship genome-wide association analyses decrease bias in estimates of direct genetic effects. Nat Genet. 2022;54:581–92. 35534559 10.1038/s41588-022-01062-7 PMC9110300

[79] Morris, T. T., Davies, N. M., Hemani, G. & Smith, G. D. *Population Phenomena Inflate Genetic Associations of Complex Social Traits*. https://www.science.org (2020). 10.1126/sciadv.aay0328 PMC7159920 32426451

[80] Young AI, et al. Mendelian imputation of parental genotypes improves estimates of direct genetic effects. Nat Genet. 2022;54:897–905. 35681053 10.1038/s41588-022-01085-0 PMC9197765

[81] Wang, Z. G. M. S. M. RNA-Seq a revolutionary tool for transcriptomics. 10.1038/nrg2484 PMC2949280 19015660

[82] Lowe R, Shirley N, Bleackley M, Dolan S, Shafee T. Transcriptomics technologies. PLoS Comput Biol. 2017;13:85. 10.1371/journal.pcbi.1005457 PMC5436640 28545146

[83] Romanoski CE, et al. Systems genetics analysis of gene-by-environment interactions in human cells. Am J Hum Genet. 2010;86:399–410. 20170901 10.1016/j.ajhg.2010.02.002 PMC2833388

[84] Orozco LD, et al. Unraveling inflammatory responses using systems genetics and gene-environment interactions in macrophages. Cell. 2012;151:658–70. 23101632 10.1016/j.cell.2012.08.043 PMC3513387

[85] Dermitzakis ET. Gene-gene and gene-environment interactions detected by transcriptome sequence analysis in twins. Nat Genet. 2015;47:88–91. 25436857 10.1038/ng.3162 PMC4643454

[86] Eid A, Mhatre I, Richardson JR. Gene-environment interactions in Alzheimer’s disease: a potential path to precision medicine. Pharmacol Therap. 2019;199:173–87. 30877021 10.1016/j.pharmthera.2019.03.005 PMC6827882

[87] Abascal F, et al. Expanded encyclopaedias of DNA elements in the human and mouse genomes. Nature. 2020;583:699–710. 32728249 10.1038/s41586-020-2493-4 PMC7410828

[88] GTEx Consortium. *The GTEx Consortium Atlas of Genetic Regulatory Effects across Human Tissues The GTEx Consortium**. www.gtexportal.org. 10.1126/science.aaz1776 PMC7737656 32913098

[89] Stricker SH, Köferle A, Beck S. From profiles to function in epigenomics. Nat Rev Genet. 2016;18:51–66. 27867193 10.1038/nrg.2016.138

[90] Mehrmohamadi M, Sepehri MH, Nazer N, Norouzi MR. A comparative overview of epigenomic profiling methods. Front Cell Dev Biol. 2021;9:26. 10.3389/fcell.2021.714687 PMC8340004 34368164

[91] Westerman KE, Sofer T. Many roads to a gene-environment interaction. Am J Hum Genet. 2024;111:626–35. 38579668 10.1016/j.ajhg.2024.03.002 PMC11023920

[92] Stepanyan A, et al. Long-term environmental metal exposure is associated with hypomethylation of CpG sites in NFKB1 and other genes related to oncogenesis. Clin Epigenetics. 2023;15:52. 37550793 10.1186/s13148-023-01536-3 PMC10405444

[93] Shiek SS, Mani MS, Kabekkodu SP, Dsouza HS. Health repercussions of environmental exposure to lead: methylation perspective. Toxicology. 2021;461:96. 10.1016/j.tox.2021.152927 34492314

[94] Choi SW, Friso S. Epigenetics: a new bridge between nutrition and health. Adv Nutr. 2010;1:8–16. 22043447 10.3945/an.110.1004 PMC3042783

[95] Kim K, Friso S, Choi SW. DNA methylation, an epigenetic mechanism connecting folate to healthy embryonic development and aging. J Nutr Biochem. 2009;20:917–26. 19733471 10.1016/j.jnutbio.2009.06.008 PMC2783701

[96] Lee HS. Impact of maternal diet on the epigenome during in utero life and the developmental programming of diseases in childhood and adulthood. Nutrients. 2015;7:9492–507. 26593940 10.3390/nu7115467 PMC4663595

[97] Singh D, et al. Hidden pharmacological activities of valproic acid: a new insight. Biomed Pharmacother. 2021;1:42. 10.1016/j.biopha.2021.112021 34463268

[98] Diederich M, Chateauvieux S, Morceau F, Dicato M. Molecular and therapeutic potential and toxicity of valproic acid. J Biomed Biotechnol. 2010;20:10. 10.1155/2010/479364 PMC2926634 20798865

[99] Grundberg E, et al. Global analysis of dna methylation variation in adipose tissue from twins reveals links to disease-associated variants in distal regulatory elements. Am J Hum Genet. 2013;93:876–90. 24183450 10.1016/j.ajhg.2013.10.004 PMC3824131

[100] Bell JT, et al. Epigenome-wide scans identify differentially methylated regions for age and age-related phenotypes in a healthy ageing population. PLoS Genet. 2012;8:285. 10.1371/journal.pgen.1002629 PMC3330116 22532803

[101] Van Dongen J, et al. Genetic and environmental influences interact with age and sex in shaping the human methylome. Nat Commun. 2016;7:52. 10.1038/ncomms11115 PMC4820961 27051996

[102] Murrell A, Rakyan VK, Beck S. From genome to epigenome. Hum Mol Genet. 2005;14:21. 10.1093/hmg/ddi110 15809270

[103] Wu YL, et al. Epigenetic regulation in metabolic diseases: mechanisms and advances in clinical study. Sig Transd Target Ther. 2023;8:56. 10.1038/s41392-023-01333-7 PMC9981733 36864020

[104] Feil R, Fraga MF. Epigenetics and the environment: emerging patterns and implications. Nat Rev Genet. 2012;13:97–109. 22215131 10.1038/nrg3142

[105] Villicaña S, Bell JT. Genetic impacts on DNA methylation: research findings and future perspectives. Genome Biol. 2021;22:56. 33931130 10.1186/s13059-021-02347-6 PMC8086086

[106] Teh AL, et al. The effect of genotype and in utero environment on interindividual variation in neonate DNA methylomes. Genome Res. 2014;24:1064–74. 24709820 10.1101/gr.171439.113 PMC4079963

[107] Gigante S, et al. Using long-read sequencing to detect imprinted DNA methylation. Nucleic Acids Res. 2019;47:74. 10.1093/nar/gkz107 PMC6486641 30793194

[108] Harper JW, Bennett EJ. Proteome complexity and the forces that drive proteome imbalance. Nature. 2016;537:328–38. 27629639 10.1038/nature19947 PMC5204264

[109] Leutert M, Entwisle SW, Villén J. Decoding post-translational modification crosstalk with proteomics. Mol Cell Proteom. 2021;20:87. 10.1016/j.mcpro.2021.100129 PMC8430371 34339852

[110] Lee JM, Hammarén HM, Savitski MM, Baek SH. Control of protein stability by post-translational modifications. Nat Commun. 2023;14:527. 36639369 10.1038/s41467-023-35795-8 PMC9839724

[111] Vileigas DF, et al. Landscape of heart proteome changes in a diet-induced obesity model. Sci Rep. 2019;9:21. 31792287 10.1038/s41598-019-54522-2 PMC6888820

[112] Félix, L. *Teratogenicity Testing Methods and Protocols Methods in Molecular Biology 1797*. http://www.springer.com/series/7651.

[113] Sinha I, et al. Changes in salivary proteome before and after cigarette smoking in smokers compared to sham smoking in nonsmokers: a pilot study. Tob Induc Dis. 2021;19:458. 10.18332/tid/138336 PMC8240953 34239408

[114] Eldjarn GH, et al. Large-scale plasma proteomics comparisons through genetics and disease associations. Nature. 2023;622:348–58. 37794188 10.1038/s41586-023-06563-x PMC10567571

[115] Sun BB, et al. Plasma proteomic associations with genetics and health in the UK Biobank. Nature. 2023;622:329–38. 37794186 10.1038/s41586-023-06592-6 PMC10567551

[116] Dhindsa RS, et al. Rare variant associations with plasma protein levels in the UK Biobank. Nature. 2023;622:339–47. 37794183 10.1038/s41586-023-06547-x PMC10567546

[117] Birhanu AG. Mass spectrometry-based proteomics as an emerging tool in clinical laboratories. Clini Proteom. 2023;20:419. 10.1186/s12014-023-09424-x PMC10464495 37633929

[118] Iwamoto N, Shimada T. Recent advances in mass spectrometry-based approaches for proteomics and biologics: great contribution for developing therapeutic antibodies. Pharmacol Ther. 2018;185:147–54. 29274706 10.1016/j.pharmthera.2017.12.007

[119] Rattray NJW, et al. Beyond genomics: understanding exposotypes through metabolomics. Hum Genom. 2018;12:215. 10.1186/s40246-018-0134-x PMC5787293 29373992

[120] Patti GJ, Yanes O, Siuzdak G. Innovation: metabolomics: the apogee of the omics trilogy. Nat Rev Mol Cell Biol. 2012;13:263–9. 22436749 10.1038/nrm3314 PMC3682684

[121] Gieger C, et al. Genetics meets metabolomics: a genome-wide association study of metabolite profiles in human serum. PLoS Genet. 2008;4:52. 10.1371/journal.pgen.1000282 PMC2581785 19043545

[122] Walker DI, et al. High-resolution metabolomics of occupational exposure to trichloroethylene. Int J Epidemiol. 2016;45:1517–27. 27707868 10.1093/ije/dyw218 PMC5100622

[123] van Veldhoven K, et al. Effects of exposure to water disinfection by-products in a swimming pool: a metabolome-wide association study. Environ Int. 2018;111:60–70. 29179034 10.1016/j.envint.2017.11.017 PMC5786667

[124] Wang, L. *et al. P A T H O L O G Y Human Genetic and Metabolite Variation Reveals That Methylthioadenosine Is a Prognostic Biomarker and an Inflammatory Regulator in Sepsis*. vol. 4 https://www.science.org. 10.1126/sciadv.1602096 PMC5342653 28345042

[125] Chu X, et al. Integration of metabolomics, genomics, and immune phenotypes reveals the causal roles of metabolites in disease. Genome Biol. 2021;22:74. 34229738 10.1186/s13059-021-02413-z PMC8259168

[126] Vineis P, et al. What is new in the exposome? Environ Int. 2020;143:569. 10.1016/j.envint.2020.105887 32619912

[127] Bononi A, et al. Heterozygous germline BLM mutations increase susceptibility to asbestos and mesothelioma. Science. 2020. 10.1073/pnas.2019652117/-/DCSupplemental. 10.1073/pnas.2019652117 PMC7776606 33318203

[128] Modafferi S, et al. Gene–environment interactions in developmental neurotoxicity: a case study of synergy between chlorpyrifos and chd8 knockout in human brainspheres. Environ Health Perspect. 2021;129:1125. 10.1289/EHP8580 PMC8278985 34259569

[129] Bakermans-Kranenburg MJ, Van Ijzendoorn MH. The hidden efficacy of interventions: gene × environment experiments from a differential susceptibility perspective. Annu Rev Psychol. 2015;66:381–409. 25148854 10.1146/annurev-psych-010814-015407

[130] Moore R, et al. A linear mixed-model approach to study multivariate gene–environment interactions. Nat Genet. 2019;51:180–6. 30478441 10.1038/s41588-018-0271-0 PMC6354905

[131] Dahl A, et al. A robust method uncovers significant context-specific heritability in diverse complex traits. Am J Hum Genet. 2020;106:71–91. 31901249 10.1016/j.ajhg.2019.11.015 PMC7042488

[132] Zhou X, Lee SH. An integrative analysis of genomic and exposomic data for complex traits and phenotypic prediction. Sci Rep. 2021;11:170. 34728654 10.1038/s41598-021-00427-y PMC8564528

[133] Motsinger-Reif AA, et al. Gene-environment interactions within a precision environmental health framework. Cell Genom. 2024;4:214. 10.1016/j.xgen.2024.100591 PMC11293590 38925123

[134] Aurich D, Miles O, Schymanski EL. Historical exposomics and high resolution mass spectrometry. Exposome. 2021;1:14.

[135] Géhin C, Holman SW. Advances in high-resolution mass spectrometry applied to pharmaceuticals in 2020: a whole new age of information. Anal Sci Adv. 2021;2:142–56. 38716455 10.1002/ansa.202000149 PMC10989654

[136] Samon SM, Hammel SC, Stapleton HM, Anderson KA. Silicone wristbands as personal passive sampling devices: current knowledge, recommendations for use, and future directions. Environ Int. 2022;169:214. 10.1016/j.envint.2022.107339 PMC9713950 36116363

[137] Koelmel JP, et al. Exploring the external exposome using wearable passive samplers-the China BAPE study. Environ Pollut. 2021;270:1459. 10.1016/j.envpol.2020.116228 33360595

[138] Jiang C, et al. Dynamic human environmental exposome revealed by longitudinal personal monitoring. Cell. 2018;175:277-291.e31. 30241608 10.1016/j.cell.2018.08.060 PMC6472932

[139] The All of Us Research Program Investigators. *The “All of Us” Research Program: Special Report*. (2019).

[140] Fayet Y, Bonnin T, Canali S, Giroux E. Putting the exposome into practice: an analysis of the promises, methods and outcomes of the European human exposome network. Soc Sci Med. 2024;354:147. 10.1016/j.socscimed.2024.117056 39029140

[141] Zhang H, et al. Genome-wide association study identifies 32 novel breast cancer susceptibility loci from overall and subtype-specific analyses. Nat Genet. 2020;52:572–81. 32424353 10.1038/s41588-020-0609-2 PMC7808397

[142] Allayee H, et al. Systems genetics approaches for understanding complex traits with relevance for human disease. eLife. 2023;12:149. 10.7554/eLife.91004 PMC10645424 37962168

[143] Roadmap Epigenomics Consortium *et al.* Integrative analysis of 111 reference human epigenomes. *Nature* **518**, 317–329 (2015). 10.1038/nature14248 PMC4530010 25693563

[144] Assum I, et al. Tissue-specific multi-omics analysis of atrial fibrillation. Nat Commun. 2022;13:119. 35064145 10.1038/s41467-022-27953-1 PMC8782899

[145] Hannon E, et al. Methylation QTLs in the developing brain and their enrichment in schizophrenia risk loci. Nat Neurosci. 2015;19:48–54. 26619357 10.1038/nn.4182 PMC4714325

[146] Trynka G, et al. Disentangling the effects of colocalizing genomic annotations to functionally prioritize non-coding variants within complex-trait Loci. Am J Hum Genet. 2015;97:139–52. 26140449 10.1016/j.ajhg.2015.05.016 PMC4572568

[147] Pickrell JK. Joint analysis of functional genomic data and genome-wide association studies of 18 human traits. Am J Hum Genet. 2014;94:559–73. 24702953 10.1016/j.ajhg.2014.03.004 PMC3980523

[148] Schaid DJ, Chen W, Larson NB. From genome-wide associations to candidate causal variants by statistical fine-mapping. Nat Rev Genet. 2018;19:491–504. 29844615 10.1038/s41576-018-0016-z PMC6050137

[149] Giambartolomei C, et al. Bayesian test for colocalisation between Pairs of genetic association studies using summary statistics. PLoS Genet. 2014;10:258. 10.1371/journal.pgen.1004383 PMC4022491 24830394

[150] He X, et al. Sherlock: detecting gene-disease associations by matching patterns of expression QTL and GWAS. Am J Hum Genet. 2013;92:667–80. 23643380 10.1016/j.ajhg.2013.03.022 PMC3644637

[151] Schwartzentruber J, et al. Genome-wide meta-analysis, fine-mapping and integrative prioritization implicate new Alzheimer’s disease risk genes. Nat Genet. 2021;53:392–402. 33589840 10.1038/s41588-020-00776-w PMC7610386

[152] Vanderweele TJ, Tchetgen Tchetgen EJ, Cornelis M, Kraft P. Methodological challenges in Mendelian randomization. Epidemiology. 2014;25:427–35. 24681576 10.1097/EDE.0000000000000081 PMC3981897

[153] Sanderson E, et al. Mendelian randomization. Nat Rev Methods Primers. 2022;2:189. 10.1038/s43586-021-00092-5 PMC7614635 37325194

[154] Richmond RC, Smith GD. Mendelian randomization: concepts and scope. Cold Spring Harb Perspect Med. 2022;12:172. 10.1101/cshperspect.a040501 PMC8725623 34426474

[155] Deng YT, et al. Identifying causal genes for depression via integration of the proteome and transcriptome from brain and blood. Mol Psychiatry. 2022;27:2849–57. 35296807 10.1038/s41380-022-01507-9

[156] Markozannes G, et al. Systematic review of Mendelian randomization studies on risk of cancer. BMC Med. 2022;20:145. 35105367 10.1186/s12916-022-02246-y PMC8809022

[157] Yuan K, et al. Fine-mapping across diverse ancestries drives the discovery of putative causal variants underlying human complex traits and diseases. Science. 2019;14:856. 10.1038/s41588-024-01870-z PMC11888783 39187616

[158] Lu Z, et al. Multi-ancestry fine-mapping improves precision to identify causal genes in transcriptome-wide association studies. Am J Hum Genet. 2022;109:1388–404. 35931050 10.1016/j.ajhg.2022.07.002 PMC9388396

[159] Wainberg M, et al. Opportunities and challenges for transcriptome-wide association studies. Nat Genet. 2019;51:592–9. 30926968 10.1038/s41588-019-0385-z PMC6777347

[160] Fan Y, et al. Multi-omics analysis reveals aberrant gut-metabolome-immune network in schizophrenia. Front Immunol. 2022;13:52. 10.3389/fimmu.2022.812293 PMC8927969 35309369

[161] Langfelder P, Horvath S. WGCNA: an R package for weighted correlation network analysis. BMC Bioinformatics. 2008;9:214. 19114008 10.1186/1471-2105-9-559 PMC2631488

[162] Sharma A, Lysenko A, Jia S, Boroevich KA, Tsunoda T. Advances in AI and machine learning for predictive medicine. J Hum Genet. 2024. 10.1038/s10038-024-01231-y. 38424184 10.1038/s10038-024-01231-y PMC11422165

[163] Camacho DM, Collins KM, Powers RK, Costello JC, Collins JJ. Next-generation machine learning for biological networks. Cell. 2018;173:1581–92. 29887378 10.1016/j.cell.2018.05.015

[164] Xu H, et al. Multi-omics marker analysis enables early prediction of breast tumor progression. Front Genet. 2021;12:18. 10.3389/fgene.2021.670749 PMC8209521 34149812

[165] Huang Q, Zhang X, Hu Z. Application of artificial intelligence modeling technology based on multi-omics in noninvasive diagnosis of inflammatory bowel disease. J Inflamm Res. 2021;14:1933–43. 34017190 10.2147/JIR.S306816 PMC8131075

[166] Malik V, Kalakoti Y, Sundar D. Deep learning assisted multi-omics integration for survival and drug-response prediction in breast cancer. BMC Genomics. 2021;22:71. 33761889 10.1186/s12864-021-07524-2 PMC7992339

[167] Wang B, et al. Similarity network fusion for aggregating data types on a genomic scale. Nat Methods. 2014;11:333–7. 24464287 10.1038/nmeth.2810

[168] Argelaguet R, et al. Multi-Omics Factor Analysis—a framework for unsupervised integration of multi-omics data sets. Mol Syst Biol. 2018;14:214. 10.15252/msb.20178124 PMC6010767 29925568

[169] Shen R, Olshen AB, Ladanyi M. Integrative clustering of multiple genomic data types using a joint latent variable model with application to breast and lung cancer subtype analysis. Bioinformatics. 2009;25:2906–12. 19759197 10.1093/bioinformatics/btp543 PMC2800366

[170] Sharifi-Noghabi, H., Zolotareva, O., Collins, C. C. & Ester, M. MOLI: Multi-omics late integration with deep neural networks for drug response prediction. in *Bioinformatics* vol. 35 i501–i509 (Oxford University Press, 2019). 10.1093/bioinformatics/btz318 PMC6612815 31510700

[171] Xu J, et al. A hierarchical integration deep flexible neural forest framework for cancer subtype classification by integrating multi-omics data. BMC Bioinformatics. 2019;20:96. 31660856 10.1186/s12859-019-3116-7 PMC6819613

[172] Drouard G, et al. Exploring machine learning strategies for predicting cardiovascular disease risk factors from multi-omic data. BMC Med Inform Decis Mak. 2024;24:49. 38698395 10.1186/s12911-024-02521-3 PMC11064347

[173] Khadirnaikar S, Shukla S, Prasanna SRM. Integration of pan-cancer multi-omics data for novel mixed subgroup identification using machine learning methods. PLoS One. 2023;18:45. 10.1371/journal.pone.0287176 PMC10586677 37856446

[174] Abbasi EY, et al. A machine learning and deep learning-based integrated multi-omics technique for leukemia prediction. Heliyon. 2024;10:46. 10.1016/j.heliyon.2024.e25369 PMC10862685 38352790

[175] Yuan M, et al. Epigenetic regulation in major depression and other stress-related disorders: molecular mechanisms, clinical relevance and therapeutic potential. Sig Transd Target Ther. 2023;8:59. 10.1038/s41392-023-01519-z PMC10465587 37644009

[176] Xie Y, et al. Integrated analysis of methylomic and transcriptomic data to identify potential diagnostic biomarkers for major depressive disorder. Genes (Basel). 2021;12:53. 10.3390/genes12020178 PMC7912210 33513891

[177] Wang C, Lue W, Kaalia R, Kumar P, Rajapakse JC. Network-based integration of multi-omics data for clinical outcome prediction in neuroblastoma. Sci Rep. 2022;12:96. 36104347 10.1038/s41598-022-19019-5 PMC9475034

[178] Vora LK, et al. Artificial intelligence in pharmaceutical technology and drug delivery design. Pharmaceutics. 2023;15:214. 37514102 10.3390/pharmaceutics15071916 PMC10385763

[179] Chekroud, A. M. *et al. The Promise of Machine Learning in Predicting Treatment Outcomes in Psychiatry*. 10.1002/wps.20882 PMC8129866 34002503

[180] Joyce JB, et al. Multi-omics driven predictions of response to acute phase combination antidepressant therapy: a machine learning approach with cross-trial replication. Transl Psychiatry. 2021;11:41. 34620827 10.1038/s41398-021-01632-z PMC8497535

[181] D.R. Cox & D. Oakes. *Analysis of Survival Data*. *Compositional Data J. Aitchison* (1984).

[182] Wu M, Huang J, Ma S. Identifying gene-gene interactions using penalized tensor regression. Stat Med. 2018;37:598–610. 29034516 10.1002/sim.7523 PMC5771864

[183] Wu M, Zhang Q, Ma S. Structured gene-environment interaction analysis. Biometrics. 2020;76:23–35. 31424088 10.1111/biom.13139 PMC7028505

[184] Liu J, et al. Identification of gene-environment interactions in cancer studies using penalization. Genomics. 2013;102:189–94. 23994599 10.1016/j.ygeno.2013.08.006 PMC3869641

[185] Bien J, Taylor J, Tibshirani R. A lasso for hierarchical interactions. Ann Stat. 2013;41:1111–41. 26257447 10.1214/13-AOS1096 PMC4527358

[186] Wu M, Ma S. Robust genetic interaction analysis. Brief Bioinform. 2019;20:624–37. 29897421 10.1093/bib/bby033 PMC6556899

[187] McAllister, K. *et al.* Current Challenges and New Opportunities for Gene-Environment Interaction Studies of Complex Diseases. in *American Journal of Epidemiology* vol. 186 753–761 (Oxford University Press, 2017). 10.1093/aje/kwx227 PMC5860428 28978193

[188] *UK Biobank: Protocol for a Large-Scale Prospective Epidemiological Resource (AMENDMENT ONE FINAL)*. (2007).

[189] Kurki MI, et al. FinnGen provides genetic insights from a well-phenotyped isolated population. Nature. 2023;613:508–18. 36653562 10.1038/s41586-022-05473-8 PMC9849126

[190] Nam, Y. *et al.* Harnessing Artificial Intelligence in Multimodal Omics Data Integration: Paving the Path for the Next Frontier in Precision Medicine. *Annual Review of Biomedical Data Science Downloaded from *www.annualreviews.org*. Guest* (2024) 10.1146/annurev-biodatasci-102523. 10.1146/annurev-biodatasci-102523-103801 PMC11972123 38768397

[191] Shuni WXYQZSM. Gene–environment interaction analysis via deep learning. Gen Epidemiol. 2023;5:693. 10.1002/gepi.22518 PMC10244912 36807383

[192] Madhukar NS, et al. A Bayesian machine learning approach for drug target identification using diverse data types. Nat Commun. 2019;10:412. 31745082 10.1038/s41467-019-12928-6 PMC6863850

[193] Sun N, Wang Y, Chu J, Han Q, Shen Y. Bayesian approaches in exploring gene-environment and gene-gene interactions: a comprehensive review. Cancer Genom Proteomics. 2023;20:669–78. 10.21873/cgp.20414 PMC10687732 38035701

[194] Zou F, Huang H, Lee S, Hoeschele I. Nonparametric Bayesian variable selection with applications to multiple quantitative trait loci mapping with epistasis and gene-environment interaction. Genetics. 2010;186:385–94. 20551445 10.1534/genetics.109.113688 PMC2940302

[195] Spanbauer C, Sparapani R. Nonparametric machine learning for precision medicine with longitudinal clinical trials and Bayesian additive regression trees with mixed models. Stat Med. 2021;40:2665–91. 33751659 10.1002/sim.8924

[196] Alsentzer, E. *et al.* Publicly Available Clinical BERT Embeddings. (2019).

[197] Lee J, et al. BioBERT: a pre-trained biomedical language representation model for biomedical text mining. Bioinformatics. 2020;36:1234–40. 31501885 10.1093/bioinformatics/btz682 PMC7703786

[198] Li, C. *et al. LLaVA-Med: Training a Large Language-and-Vision Assistant for Biomedicine in One Day*. https://aka.ms/llava-med.

[199] Zuo, X., Yang, X., Dou, Z. & Wen, J. R. MedGPT: Medical Concept Prediction from Clinical Narratives. in *28th Text REtrieval Conference, TREC 2019-Proceedings* (National Institute of Standards and Technology (NIST), 2019). 10.1145/1122445.1122456.

[200] Obermeyer, Z., Powers, B., Vogeli, C. & Mullainathan, S. *Dissecting Racial Bias in an Algorithm Used to Manage the Health of Populations*. https://www.science.org. 10.1126/science.aax2342 31649194

[201] Rudin C. Stop explaining black box machine learning models for high stakes decisions and use interpretable models instead. Nat Mach Intell. 2019;1:206–15. 35603010 10.1038/s42256-019-0048-x PMC9122117

[202] Dwork C, Roth A. The algorithmic foundations of differential privacy. Found Trends Theor Comput Sci. 2013;9:211–487.

[203] Mills MC, Rahal C. The GWAS Diversity Monitor tracks diversity by disease in real time. Nat Genet. 2020;2:963. 10.1038/s41588-020-0580-y 32139905

[204] Privé F, et al. Portability of 245 polygenic scores when derived from the UK Biobank and applied to 9 ancestry groups from the same cohort. Am J Hum Genet. 2022;109:12–23. 34995502 10.1016/j.ajhg.2021.11.008 PMC8764121

[205] Metspalu M, et al. Shared and unique components of human population structure and genome-wide signals of positive selection in South Asia. Am J Hum Genet. 2011;89:731–44. 22152676 10.1016/j.ajhg.2011.11.010 PMC3234374

[206] Gomez F, Hirbo J, Tishkoff SA. Genetic variation and adaptation in Africa: Implications for human evolution and disease. Cold Spring Harb Perspect Biol. 2014;6:13. 10.1101/cshperspect.a008524 PMC4067985 24984772

[207] Genovese G, et al. A risk allele for focal segmental glomerulosclerosis in African Americans is located within a region containing APOL1 and MYH9. Kidney Int. 2010;78:698–704. 20668430 10.1038/ki.2010.251 PMC3001190

[208] Rotimi CN, et al. The genomic landscape of African populations in health and disease. Hum Mol Genet. 2017;26:225–36. 10.1093/hmg/ddx253 PMC6075021 28977439

[209] Cohen J, et al. Low LDL cholesterol in individuals of African descent resulting from frequent nonsense mutations in PCSK9. Nat Genet. 2005;37:161–5. 15654334 10.1038/ng1509

[210] Breeze CE, Beck S, Berndt SI, Franceschini N. The missing diversity in human epigenomic studies. Nat Genet. 2022;54:737–9. 35681055 10.1038/s41588-022-01081-4 PMC9832920

[211] Dlamini SN, et al. Associations Between CYP17A1 and SERPINA6/A1 polymorphisms, and cardiometabolic risk factors in Black South Africans. Front Genet. 2021;12:148. 10.3389/fgene.2021.687335 PMC8414563 34484290

[212] Rotimi C, et al. Research capacity. Enabling the genomic revolution in Africa. Science. 2014;344:1346–8. 24948725 10.1126/science.1251546 PMC4138491

[213] Aron S, et al. The development of a sustainable bioinformatics training environment within the H3Africa bioinformatics network (H3ABioNet). Front Educ (Lausanne). 2021;6:459.

[214] Chen C, et al. Applications of multi-omics analysis in human diseases. MedComm. 2023;4:63. 10.1002/mco2.315 PMC10390758 37533767

[215] Krassowski M, Das V, Sahu SK, Misra BB. State of the field in multi-omics research: from computational needs to data mining and sharing. Front Genet. 2020;11:52. 33362867 10.3389/fgene.2020.610798 PMC7758509

[216] *Bioinformatics Methods in Clinical Research*. vol. 593 (Humana Press, Totowa, 2010).

[217] Prosperi M, Min JS, Bian J, Modave F. Big data hurdles in precision medicine and precision public health. BMC Med Inform Decis Mak. 2018;18:214. 10.1186/s12911-018-0719-2 PMC6311005 30594159

[218] Kreitmaier P, Katsoula G, Zeggini E. Insights from multi-omics integration in complex disease primary tissues. Trends Genet. 2023;39:46–58. 36137835 10.1016/j.tig.2022.08.005

[219] Du Y, Fan K, Lu X, Wu C. Integrating multi-omics data for gene-environment interactions. BioTech. 2021;10:56. 10.3390/biotech10010003 PMC9245467 35822775

[220] Kanai M, et al. Meta-analysis fine-mapping is often miscalibrated at single-variant resolution. Cell Genom. 2022;2:459. 10.1016/j.xgen.2022.100210 PMC9839193 36643910

[221] Lusis AJ, et al. The hybrid mouse diversity panel: a resource for systems genetics analyses of metabolic and cardiovascular traits. J Lipid Res. 2016;57:925–42. 27099397 10.1194/jlr.R066944 PMC4878195

[222] Hayman, G. T., Smith, J. R. & Dwinell, M. R. *Rat Genomics*. http://www.springer.com/series/7651.

[223] MacKay TFC, et al. The drosophila melanogaster genetic reference panel. Nature. 2012;482:173–8. 22318601 10.1038/nature10811 PMC3683990

[224] Le Goff A, Louvel S, Boullier H, Allard P. Toxicoepigenetics for risk assessment: bridging the gap between basic and regulatory science. Epigenetics Insights. 2022;15:214. 10.1177/25168657221113149 PMC9290111 35860623

[225] Hsu L, et al. Powerful cocktail methods for detecting genome-wide gene-environment interaction. Genet Epidemiol. 2012;36:183–94. 22714933 10.1002/gepi.21610 PMC3654520

[226] David W. Threadgill, Darla R. Miller, Gary A. Churchill & Fernando Pardo-Manuel de Villena. The Collaborative Cross-A Recombinant Inbred Mouse Population for the Systems Genetic Era. 10.1093/ilar.52.1.24 21411855

[227] Bogue MA, Churchill GA, Chesler EJ. Collaborative cross and diversity outbred data resources in the mouse phenome database. Mammalian Genome. 2015;26:511–20. 26286858 10.1007/s00335-015-9595-6 PMC4602074

[228] Cheung M, Testa JR. BAP1, a tumor suppressor gene driving malignant mesothelioma. Transl Lung Cancer Res. 2017;6:270–8. 28713672 10.21037/tlcr.2017.05.03 PMC5504107

[229] Akhtari FS, et al. High-throughput screening and genome-wide analyses of 44 anticancer drugs in the 1000 Genomes cell lines reveals an association of the NQO1 gene with the response of multiple anticancer drugs. PLoS Genet. 2021;17:245. 10.1371/journal.pgen.1009732 PMC8439493 34437536

[230] Balik-Meisner M, et al. Elucidating gene-by-environment interactions associated with differential susceptibility to chemical exposure. Environ Health Perspect. 2018;126:410. 10.1289/EHP2662 PMC6084885 29968567

[231] Wu H, Eckhardt CM, Baccarelli AA. Molecular mechanisms of environmental exposures and human disease. Nat Rev Genet. 2023;24:332–44. 36717624 10.1038/s41576-022-00569-3 PMC10562207

[232] Perlman RL. Mouse models of human disease: an evolutionary perspective. Evol Med Public Health. 2016;2:14. 10.1093/emph/eow014 PMC4875775 27121451

[233] Ritchie, M. D. *et al.* Incorporation of Biological Knowledge into the Study of Gene-Environment Interactions. in *American Journal of Epidemiology* vol. 186, pp 771–777 (Oxford University Press, 2017). 10.1093/aje/kwx229 PMC5860556 28978191

[234] Dunham I, et al. An integrated encyclopedia of DNA elements in the human genome. Nature. 2012;489:57–74. 22955616 10.1038/nature11247 PMC3439153

[235] The GTEx Consortium. *The Genotype-Tissue Expression (GTEx) Pilot Analysis: Multitissue Gene Regulation in Humans*. https://www.science.org. 10.1126/science.1262110 PMC4547484 25954001

[236] Shalem O, Sanjana NE, Zhang F. High-throughput functional genomics using CRISPR-Cas9. Nat Rev Genet. 2015;16:299–311. 25854182 10.1038/nrg3899 PMC4503232

[237] Tian R, et al. Genome-wide CRISPRi/a screens in human neurons link lysosomal failure to ferroptosis. Nat Neurosci. 2021;24:1020–34. 34031600 10.1038/s41593-021-00862-0 PMC8254803

[238] Baye TM, Abebe T, Wilke RA. Genotype-environment interactions and their translational implications. Personal Med. 2011;8:59–70. 10.2217/pme.10.75 PMC3108095 21660115

[239] Elbaz A, et al. CYP2D6 polymorphism, pesticide exposure, and Parkinson’s disease. Ann Neurol. 2004;55:430–4. 14991823 10.1002/ana.20051

[240] Lewis SJ, Smith GD. Alcohol, ALDH2, and esophageal cancer: a meta-analysis which illustrates the potentials and limitations of a Mendelian randomization approach. Cancer Epidemiol Biomark Prev. 2005;14:1967–71. 10.1158/1055-9965.EPI-05-0196 16103445

[241] The International Warfarin Pharmacogenetics Consortium. Estimation of the Warfarin dose with clinical and pharmacogenetic data. N Engl J Med. 2009;360:753–64. 19228618 10.1056/NEJMoa0809329 PMC2722908

[242] Zhou S. Clinical pharmacogenomics of thiopurine S-methyltransferase. Curr Clini Pharmacol. 2006;1:258. 10.2174/157488406784111627 18666383

[243] Olopade OI, Grushko TA, Nanda R, Huo D. Advances in breast cancer: pathways to personalized medicine. Clini Cancer Res. 2008;14:7988–99. 10.1158/1078-0432.CCR-08-1211 PMC4535810 19088015

[244] McAllister, K. *et al.* Current Challenges and New Opportunities for Gene-Environment Interaction Studies of Complex Diseases. in *American Journal of Epidemiology* vol. 186 753–761 (Oxford University Press, 2017). 10.1093/aje/kwx227 PMC5860428 28978193

[245] Gref A, et al. Genome-wide interaction analysis of air pollution exposure and childhood asthma with functional follow-up. Am J Respir Crit Care Med. 2017;195:1373–83. 27901618 10.1164/rccm.201605-1026OC PMC5443897

[246] Bentley AR, et al. Multi-ancestry genome-wide gene–smoking interaction study of 387,272 individuals identifies new loci associated with serum lipids. Nat Genet. 2019;51:636–48. 30926973 10.1038/s41588-019-0378-y PMC6467258

[247] Kilpeläinen TO, et al. Multi-ancestry study of blood lipid levels identifies four loci interacting with physical activity. Nat Commun. 2019;10:245. 30670697 10.1038/s41467-018-08008-w PMC6342931

[248] Ortega-Azorín C, et al. Associations of the FTO rs9939609 and the MC4R rs17782313 polymorphisms with type 2 diabetes are modulated by diet, being higher when adherence to the Mediterranean diet pattern is low. Cardiovasc Diabetol. 2012;11:89. 23130628 10.1186/1475-2840-11-137 PMC3495759

[249] Fisher E, et al. Whole-grain consumption and transcription factor-7-like 2 (TCF7L2) rs7903146: Gene-diet interaction in modulating type 2 diabetes risk. Br J Nutr. 2009;101:478–81. 19149908 10.1017/S0007114508020369

[250] Figueiredo JC, et al. Genome-wide diet-gene interaction analyses for risk of colorectal cancer. PLoS Genet. 2014;10:490. 10.1371/journal.pgen.1004228 PMC3990510 24743840

[251] Hoang T, Cho S, Choi JY, Kang D, Shin A. Genome-wide interaction study of dietary intake and colorectal cancer risk in the UK Biobank. JAMA Netw Open. 2024;7:e240465. 38411962 10.1001/jamanetworkopen.2024.0465 PMC10900970

[252] Anand A, Koller DL, Lawson WB, Gershon ES, Nurnberger JI. Genetic and childhood trauma interaction effect on age of onset in bipolar disorder: an exploratory analysis. J Affect Disord. 2015;179:1–5. 25837715 10.1016/j.jad.2015.02.029 PMC5845791

[253] Dunn EC, et al. Genome-wide association study (GWAS) and genome-wide by environment interaction study (GWEIS) of depressive symptoms in African American and Hispanic/Latina Women. Depress Anxiety. 2016;33:265–80. 27038408 10.1002/da.22484 PMC4826276

[254] Vaucher J, et al. Cannabis use and risk of schizophrenia: a Mendelian randomization study. Mol Psychiatry. 2018;23:1287–92. 28115737 10.1038/mp.2016.252 PMC5984096

[255] Schaffner SL, et al. Genetic variation and pesticide exposure influence blood DNA methylation signatures in females with early-stage Parkinson’s disease. NPJ Parkinsons Dis. 2024;10:120. 38714693 10.1038/s41531-024-00704-3 PMC11076573

[256] Ngo KJ, et al. Lysosomal genes contribute to Parkinson’s disease near agriculture with high intensity pesticide use. NPJ Parkinsons Dis. 2024;10:149. 38664407 10.1038/s41531-024-00703-4 PMC11045791

