# Supplementary material for: Multi-omics approaches for understanding gene-environment interactions in noncommunicable diseases: techniques, translation, and equity issues
Source: Hum Genomics. 2025 Jan 31;19:8. doi: 10.1186/s40246-025-00718-9 (PMC11786457; doi:10.1186/s40246-025-00718-9)
Supplement: Supplementary file 3 — Additional file 3. [file 40246_2025_718_MOESM3_ESM.docx]

**Supplementary Information**

**Table S1**. List of example gene-environment interactions in NCDs

| **Genes** | **Environmental exposure** | **Phenotype** | **SNP** | **PMID** |
| --- | --- | --- | --- | --- |
| *DLG2* | Air pollution | Asthma | rs963146 | 27901618 |
| *B4GALT5* | Air pollution | Asthma | rs686237 | 27901618 |
| *ADCY2* | Air pollution | Asthma | rs6886921, rs727432, rs4143882 | 27901618 |
| *LHX1* | Physical activity | Coronary artery disease | rs295849 | 30670697 |
| *CLASP1* | Physical activity | Coronary artery disease | rs2862183 | 30670697 |
| *CNTNAP2* | Physical activity | Coronary artery disease | rs190748049 | 30670697 |
| *EPDR1* | Fish oil intake | Colorectal cancer | rs2709110, rs2718059, rs1450847, rs35202238, rs1597550, and rs1376256 | 38411962 |
| *GATA3* | Processed meat | Colorectal cancer | rs4143094 | 24743840 |
| *GBA1* | Pesticides | Parkinson's disease | rs75822236 | 38664407 |
| *NFATC* | Pesticides | Parkinson's disease | chr6:27569167–27570549 such as rs1214587443, rs1338028325, rs1317161688, rs1407154306, rs552610347 | 38714693 |
| *EYA1* | Smoking | Coronary artery disease | rs10101067 | 30926973 |
| *TMEM175* | Smoking | Coronary artery disease | rs34311866 | 30926973 |
| *ETV5* | Smoking | Coronary artery disease | rs10937241 | 30926973 |
| *KCNT2* | Cannabis use | Schizophrenia | rs73067624 | 28115737* |
| *NCAM1* | Cannabis use | Schizophrenia | rs4471463 | 28115737* |
| *CEP350* | Stressful life events | Depression | rs4652467 | 27038408 |
| *CACNA2D4* | Childhood maltreatment | Bipolar disorder | rs12368779 | 25837715 |
| *TCF7L2* | Whole-grain intake | Type 2 diabetes | rs7903146 | 19149908 |
| *IGF2BP2* | Dietary fat intake | Type 2 diabetes | rs4402960 | 20215779 |
| *FTO* | Mediterranean diet | Type 2 diabetes | rs9939609 | 23130628 |
| *MC4R* | Mediterranean diet | Type 2 diabetes | rs17782313 | 23130628 |

Notes: Most of the gene-environment interaction results summarized in this table are from genome-wide environmental interaction studies, while two stem from Mendelian randomization analyses. Variants in *DLG2*, *ADCY1/2*, and *B4GALT5* interacting with air pollutants are associated with higher asthma susceptibility^245^; *TMEM175*, *ETV5*, and *EYA1* variants interacting with smoking increase coronary artery disease risk^246^, whereas *CLASP1*, *LHX1*, and *CNTNAP2* variants linked to higher physical activity reduce it^247^. *FTO* and *MC4R* variants interacting with a Mediterranean diet^248^ and *TCF7L2* variant with whole-grain intake reduce type 2 diabetes risk^249^ , while *IGF2BP2* variants interacting with dietary fat intake increase it^249^. *GATA3* variant interacting with processed meat intake increases colorectal cancer risk^250^, whereas *EPDR1* variants interacting with fish oil intake reduce it^251^. *CACNA2D4* variants interacting with childhood maltreatment are associated with bipolar disorder risk^252^; *CEP350* variants interacting with stressful life events elevate major depression risk^253^; *NCAM1* and *KCNT2* variants interacting with cannabis use are linked to higher schizophrenia risk^254^; and *NFATC* and *GBA1* variants interacting with agricultural pesticides increase susceptibility to Parkinson’s disease^255,256^. Studies using candidate-gene approaches were excluded due to inherent limitations such as high risk of false positives, lower replication rates, selection bias, and limited genetic coverage.

**Table S2.** A non-exhaustive list of tools for multi-omics data integration and GxE interaction analyses

| **Tool** | **Description** | **Interface** | **Omics** | **Reference** |
| --- | --- | --- | --- | --- |
| **Enrichment-Based** |  |  |  |  |
| DEPICT (Data-driven Expression Prioritized Integration for Complex Traits) | Integrative tool that employs predicted gene functions to systematically prioritize the most likely causal genes at associated loci, highlight enriched pathways and identify tissues/cell types where genes from associated loci are highly expressed. | Python | GWAS SNPs, Gene Expression | Pers et al. (2015)^14^; Watanabe et al. (2017)^15^ |
| fGWAS | Command line tool for integrating functional genomic information into a genome-wide association study (GWAS) | Command line | GWAS SNPs, Functional Annotations | Pickrell, 2014^16^ |
| FUMA (Functional Mapping and Annotation) | A platform that can be used to annotate, prioritize, visualize and interpret GWAS results. | Web-based | GWAS SNPs, Functional Annotations | Watanabe et al. (2017)^15^ |
| GARFIELD (GWAS analysis of regulatory or functional information enrichment with LD correction) | Leverages GWAS findings with functional (that is, regulatory or protein-coding) annotations to find features relevant to a phenotype of interest. It accounts for LD, matched genotyping variants and local gene density with the application of logistic regression to derive statistical significance. | R | GWAS SNPs, Functional Annotations | Iotchkova et al. (2019)^17^ |
| PascalX (Pathway scoring algorithm) | A tool for computing gene and pathway scores from SNP-phenotype association summary statistics; without the need to access the original genotypic data. | Python | GWAS SNPs | Krefl et al. (2023)^18^ |
|  |  |  |  |  |
| **Statistical Fine-Mapping** |  |  |  |  |
| Coloc (Colocalisation Tests of Two Genetic Traits) | Bayesian Test for Colocalisation between Pairs of Genetic Association Studies Using Summary Statistics. It is a web-based designed for integration of GWAS results using only p-values and the sample size of the datasets | R | Colocalization | Giambartolomei et al. (2014)^19^ |
| epiCOLOC | A web potal for integrating large-scale and context-dependent epigenomics eeatures for comprehensive colocalization analysis. The portal integrated over 44,385 bulk or single-cell epigenomic assays across 53 human tissues/cell types, such as transcription factor binding, histone modification, open chromatin and transcriptional event. | Web-based | Colocalization for epigenomic variants | Zhou et al. (2020)^20^ |
| HyPrColoc(Hypothesis Prioritisation for multi-trait Colocalization) | A deterministic Bayesian algorithm using GWAS summary statistics that can detect colocalization across vast numbers of traits simultaneously (e.g. 100 traits can be jointly analysed in around 1 s). | R | Colocalization for multiple traits | Foley et al. (2021)^21^ |
| SMR/HEIDI | Integrates summary-level data from GWAS with data from expression quantitative trait locus (eQTL) studies to identify genes whose expression levels are associated with a complex trait because of pleiotropy. This tool can therefore be used to prioritize genes underlying GWAS hits for follow-up functional studies. The approach is applicable to all kinds of molecular QTL (xQTL) data, including DNA methylation QTL (mQTL) and protein abundance QTL (pQTL) | Linux based Web-based | Mendelian Randomization | Zhu et al. (2016)^22^ |
|  |  |  |  |  |
| **Imputation-Based Methods** |  |  |  |  |
| EpiXcan | A method that increases prediction accuracy in transcriptome imputation by integrating epigenetic data to model the prior probability that a SNP affects transcription. EpiXcan specifically leverages annotations derived from the Roadmap Epigenomics Mapping Consortium (REMC) that integrates multiple epigenetic assays, including DNA methylation, histone modification and chromatin accessibility | Linux-HPC Python | GWAS SNPs, Gene Expression, DNA Methylation | Gusev et al. (2016)^23^; Zhang et al. (2019)^24^ |
| EstiMeth (Genetic estimators of epigenetic regulation) | A tool that can identify methylation-related genomic loci associated with polygenic traits and common diseases. | Python/R | GWAS SNPs, DNA Methylation | Freytag et al. (2018)^25^ |
| FUSION | A suite of tools for performing transcriptome-wide and regulome-wide association studies (TWAS and RWAS). FUSION builds predictive models of the genetic component of a functional/molecular phenotype and predicts and tests that component for association with disease using GWAS summary statistics. The goal is to identify associations between a GWAS phenotype and a functional phenotype that was only measured in reference data. | R/Python | GWAS SNPs, Gene Expression | Gusev et al. (2016)^23^ |
| MOSTWAS (Multi-Omic Strategies for TWAS) | a suite of tools that extends the TWAS framework to include no only variants that are close to a gene but also other variants far away from the gene that may be involved in complex regulatory mechanisms. It leverages multi-omic data of regulatory biomarkers (transcription factors, microRNAs, epigenetics) and borrows from techniques in mediation analysis to prioritize distal variants that are around these regulatory biomarkers. | R | GWAS SNPs, Gene Expression, DNA Methylation | Bhattacharya et al. (2021)^26^ |
| S-PrediXcan | A gene-level association approach that tests the mediating effects of gene expression levels on phenotypes. PrediXcan is implemented on GWAS or sequencing studies (i.e., studies with genome-wide interrogation of DNA variation and phenotypes). It imputes transcriptome levels with models trained in measured transcriptome datasets (e.g., GTEx). These predicted expression levels are then correlated with the phenotype in a gene association test that addresses some of the key limitations of GWAS | Linux  Python | GWAS SNPs, Gene Expression | Barbeira et al. (2018)^27^ |
| **GxE Detection Methods** |  |  |  |  |
| GEM (Gene–Environment interaction analysis in Millions of samples) | A software tool for genome-wide gene-environment (GxE) interaction analysis, designed for large-scale datasets. GEM implements a generalized linear model with robust standard errors, enabling analysis of continuous and binary phenotypes while adjusting for confounding effects. Its computational approach leverages matrix projection to reduce redundant calculations, ensuring scalability with sample size. Multi-threading via the C++ Boost library enhances efficiency, and its implementation supports seamless use across diverse environments through Docker images and cloud-computing workflows. | Linux, C++ (library dependencies) | GWAS SNPs, environmental exposures (e.g. sex, age, BMI) | Westerman et al. (2021)^28^ |
| SUGEN | A command-line software package for association analysis under complex survey sampling. SUGEN supports continuous, binary, and right-censored time-to-event traits. It performs single-variant (standard, conditional, and gene-environment interaction) and gene-based association analyses using regression models. For gene-based analysis, SUGEN outputs score statistics and covariance matrices, which can be integrated with MASS for further testing. | C++ | GWAS SNPs, Environmental Exposure | Lin et al. (2014)^29^ |
| MST (Multivariate Scale Test) | A statistical tool for detecting gene-environment (GxE) interactions across multiple continuous traits. MST integrates multiple traits into its analysis to increase power, reducing the multiple-testing burden seen in univariate methods. It does not require prior specification of environmental factors, making it suitable for exploratory GxE studies. MST has been applied to large datasets, such as the Taiwan Biobank, to identify variance quantitative trait loci (vQTLs) and uncover novel GxE signals. | R | GWAS SNPs, Environmental Exposure | Lin et al. (2024)^30^ |
| MTAGEI (Multi-Trait Analysis of Gene–Environment Interactions) | A robust and computationally efficient framework for detecting gene–environment interactions (GEI) across multiple traits and variants in large-scale genetic studies. The tool supports summary statistics-based analysis, allowing meta-analyses of consortium-based studies. By aggregating GEI signals across traits and variants, MTAGEI enhances detection power while remaining robust to diverse genetic architectures. Its omnibus approach combines single- and multi-trait tests with single- and multi-variant tests to accommodate various patterns of GEI signals. Applications include GEI analysis in the UK Biobank, where it identified novel gene-sex interactions influencing lipid traits. MTAGEI achieves superior computational efficiency with parallel processing and advanced statistical methodologies. | R  Web-based | SNPs, Environmental Exposures | Luo et al. (2024)^31^ |
| MPGE (Multi-Phenotype Gene–Environment Interaction Analysis) | An R package designed to enhance the power of gene–environment interaction (GxE) analysis by simultaneously analyzing multiple phenotypes through a two-step approach. It combines the benefits of multivariate phenotype evaluation and sequential filtering of less relevant genetic variants. MPGE first identifies promising SNPs with marginal genetic effects across multiple traits and then tests for aggregate-level GxE effects on these traits. By leveraging pleiotropy in both genetic main effects and GxE effects, MPGE demonstrates significant power gains over conventional single-phenotype or one-step approaches. The package has been applied to genome-wide GxE analysis in the UK Biobank, identifying novel loci for lipid traits influenced by alcohol consumption. | R | SNPs, Environmental Exposures | Majumdar et al. (2021)^32^ |
| LEMMA (Linear Environment Mixed Model Analysis) | A Bayesian whole-genome regression tool designed to model gene-environment (GxE) interactions in large-scale datasets, such as the UK Biobank. The method introduces an interpretable environmental score (ES), a linear combination of multiple environmental variables, to assess how genetic variants interact with combined environmental exposures. LEMMA estimates the proportion of phenotypic variance attributable to GxE effects and conducts genome-wide interaction testing for both genotyped and imputed SNPs. Robust standard errors address heteroskedasticity caused by GxE effects, and the software is computationally efficient, leveraging MoG priors and variational inference. | C++ | SNPs, Environmental Exposures | Kerin and Marchini (2020)^33^ |
|  |  |  |  |  |
| **Machine Learning/AI-Based** |  |  |  |  |
| mixOmics | An R package which classifies sample groups, identifies discriminant features, and predicts the class of new samples. By adopting a systems biology approach, the toolkit provides a wide range of methods that statistically integrate several data sets at once to probe relationships between heterogeneous ‘omics data sets. | R | Handles molecular features measured on a continuous scale (e.g. microarray, mass spectrometry-based proteomics and metabolomics) or sequenced-based count data (RNA-seq, 16S, shotgun metagenomics) that become “continuous” data after pre-processing and normalization | Rohart et al. (2017)^34^ |
| MOFA (Multi‐Omics Factor Analysis) | A statistical approach for integrating multi-omics datasets in an unsupervised manner. The method infers a set of (hidden) factors that capture biological and technical sources of variability. It disentangles axes of heterogeneity that are shared across multiple modalities and those specific to individual data modalities. The learnt factors enable a variety of downstream analyses, including identification of sample subgroups, data imputation and the detection of outlier samples. | R | Normalized multiple omics datasets | Argelaguet et al. (2018)^35^ |
| MCIA (multiple co-inertia analysis) | A multivariate analysis method that investigates the relationship patterns in multi-omics datasets - uses both sparse and structured approaches. It simultaneously projects several datasets into the same dimensional space, transforming diverse sets of features onto the same scale, to extract the most variant from each dataset and facilitate biological interpretation and pathway analysis using covariance optimization criterion. | R | Normalized multiple omics datasets | Meng et al. (2014)^36^ |
| MOGONET(Multi-Omics Graph cOnvolutional NETworks) | Jointly explores omics-specific learning and cross-omics correlation learning for effective multi-omics data classification. It can identify important biomarkers from different omics data types related to the investigated biomedical problems. | Python | mRNA expression data, DNA methylation data, and microRNA expression data | Wang et al. (2021)^37^ |
| ClustOmics | Relies on a non-relational graph database, which allows for the simultaneous integration of both multiple omics data and results from various clustering methods. tool conciliates input clusterings, regardless of their origin, their number, their size or their shape. ClustOmics implements an intuitive and flexible strategy, based upon the idea of evidence accumulation clustering. It computes co-occurrences of pairs of samples in input clusters and uses this score as a similarity measure to reorganize data into consensus clusters. | Python | Gene expression, miRNA expression and methylation | Brière et al. (2021)^38^ |
| DeepProg | Ensemble framework of deep-learning and machine-learning approaches that robustly predicts patient survival subtypes using multi-omics data. It identifies two optimal survival subtypes in most cancers and yields significantly better risk-stratification than other multi-omics integration methods. | Python | RNA-Seq, MicroRNA and Methylation | Poirion et al. (2021)^39^ |
| RWRF (Random Walk with Restart for multi-dimensional data Fusion) | A multi-omics data integration algorithm based on random walk with restart (RWR) on multiplex network. It uses similarity network of samples as the basis for integration and constructs the similarity network for each data type and then connects corresponding samples of multiple similarity networks to create a multiplex sample network. | Web-based | mRNA expression, DNA methylation, and microRNA (miRNA) expression) | Wen et al. (2021)^40^ |
| NetICS (Network-based Integration of Multi-omics Data) | A graph diffusion-based method for prioritizing cancer genes by integrating diverse molecular data types on a directed functional interaction network. NetICS prioritizes genes by their mediator effect, defined as the proximity of the gene to upstream aberration events and to downstream differentially expressed genes and proteins in an interaction network. Genes are prioritized for individual samples separately and integrated using a robust rank aggregation technique. NetICS provides a comprehensive computational framework that can aid in explaining the heterogeneity of aberration events by their functional convergence to common differentially expressed genes and proteins. | MATLAB | Ranks mediator genes for each cancer | Dimitrakopoulos et al. (2018)^41^ |
| CoSpar (Coherent sparse optimization) | A computational approach to infer cell dynamics from single-cell transcriptomics integrated with lineage tracing. Built on assumptions of coherence and sparsity of transition maps, CoSpar is robust to severe downsampling and dispersion of lineage data, which enables simpler experimental designs and requires less calibration | Python | Single-cell RNA sequencing (scRNA-seq) data | Wang et al. (2022)^42^ |
| DeepGE-MCP | A deep learning model designed to enhance feature representation for gene-environment (GxE) interaction analysis by integrating matching, correlation, and prediction subnets. These subnets capture relationships among genomic features, environmental factors, and outcomes, improving prediction accuracy and representation power. | Python | Gene-Expression (RNA-seq); Environment (age, gender, smoking pack years, tumor stage) | Wu et al. (2022)^43^ |

Notes: The table categorizes tools based on their methodology, including enrichment-based approaches, statistical fine-mapping, imputation-based methods, GxE detection methods, and machine learning/artificial intelligence (AI)-based techniques. Each entry includes a brief description of the tool, its interface type, the omics data it supports, and the relevant reference for further details.

**Supplementary references**

1. Gref A, Merid SK, Gruzieva O, et al. Genome-wide interaction analysis of air pollution exposure and childhood asthma with functional follow-up. *American journal of respiratory and critical care medicine* 2017; **195**(10): 1373-83.

2. Bentley AR, Sung YJ, Brown MR, et al. Multi-ancestry genome-wide gene-smoking interaction study of 387,272 individuals identifies new loci associated with serum lipids. *Nat Genet* 2019; **51**(4): 636-48.

3. Kilpeläinen TO, Bentley AR, Noordam R, et al. Multi-ancestry study of blood lipid levels identifies four loci interacting with physical activity. *Nat Commun* 2019; **10**(1): 376.

4. Ortega-Azorín C, Sorlí JV, Asensio EM, et al. Associations of the FTO rs9939609 and the MC4R rs17782313 polymorphisms with type 2 diabetes are modulated by diet, being higher when adherence to the Mediterranean diet pattern is low. *Cardiovasc Diabetol* 2012; **11**: 137.

5. Fisher E, Boeing H, Fritsche A, Doering F, Joost HG, Schulze MB. Whole-grain consumption and transcription factor-7-like 2 ( TCF7L2) rs7903146: gene-diet interaction in modulating type 2 diabetes risk. *Br J Nutr* 2009; **101**(4): 478-81.

6. Ruchat SM, Elks CE, Loos RJ, et al. Evidence of interaction between type 2 diabetes susceptibility genes and dietary fat intake for adiposity and glucose homeostasis-related phenotypes. *J Nutrigenet Nutrigenomics* 2009; **2**(4-5): 225-34.

7. Figueiredo JC, Hsu L, Hutter CM, et al. Genome-wide diet-gene interaction analyses for risk of colorectal cancer. *PLoS Genet* 2014; **10**(4): e1004228.

8. Hoang T, Cho S, Choi JY, Kang D, Shin A. Genome-Wide Interaction Study of Dietary Intake and Colorectal Cancer Risk in the UK Biobank. *JAMA Netw Open* 2024; **7**(2): e240465.

9. Anand A, Koller DL, Lawson WB, Gershon ES, Nurnberger JI. Genetic and childhood trauma interaction effect on age of onset in bipolar disorder: An exploratory analysis. *J Affect Disord* 2015; **179**: 1-5.

10. Dunn EC, Wiste A, Radmanesh F, et al. GENOME-WIDE ASSOCIATION STUDY (GWAS) AND GENOME-WIDE BY ENVIRONMENT INTERACTION STUDY (GWEIS) OF DEPRESSIVE SYMPTOMS IN AFRICAN AMERICAN AND HISPANIC/LATINA WOMEN. *Depress Anxiety* 2016; **33**(4): 265-80.

11. Vaucher J, Keating BJ, Lasserre AM, et al. Cannabis use and risk of schizophrenia: a Mendelian randomization study. *Mol Psychiatry* 2018; **23**(5): 1287-92.

12. Schaffner SL, Casazza W, Artaud F, et al. Genetic variation and pesticide exposure influence blood DNA methylation signatures in females with early-stage Parkinson's disease. *NPJ Parkinsons Dis* 2024; **10**(1): 98.

13. Ngo KJ, Paul KC, Wong D, et al. Lysosomal genes contribute to Parkinson's disease near agriculture with high intensity pesticide use. *NPJ Parkinsons Dis* 2024; **10**(1): 87.

14. Pers TH, Karjalainen JM, Chan Y, et al. Biological interpretation of genome-wide association studies using predicted gene functions. *Nature Communications* 2015; **6**(1): 5890.

15. Watanabe K, Taskesen E, van Bochoven A, Posthuma D. Functional mapping and annotation of genetic associations with FUMA. *Nature Communications* 2017; **8**(1): 1826.

16. Pickrell Joseph K. Joint Analysis of Functional Genomic Data and Genome-wide Association Studies of 18 Human Traits. *The American Journal of Human Genetics* 2014; **94**(4): 559-73.

17. Iotchkova V, Ritchie GRS, Geihs M, et al. GARFIELD classifies disease-relevant genomic features through integration of functional annotations with association signals. *Nat Genet* 2019; **51**(2): 343-53.

18. Krefl D, Brandulas Cammarata A, Bergmann S. PascalX: a Python library for GWAS gene and pathway enrichment tests. *Bioinformatics* 2023; **39**(5).

19. Giambartolomei C, Vukcevic D, Schadt EE, et al. Bayesian test for colocalisation between pairs of genetic association studies using summary statistics. *PLoS Genet* 2014; **10**(5): e1004383.

20. Zhou Y, Sun Y, Huang D, Li MJ. epiCOLOC: Integrating Large-Scale and Context-Dependent Epigenomics Features for Comprehensive Colocalization Analysis. *Front Genet* 2020; **11**: 53.

21. Foley CN, Staley JR, Breen PG, et al. A fast and efficient colocalization algorithm for identifying shared genetic risk factors across multiple traits. *Nat Commun* 2021; **12**(1): 764.

22. Zhu Z, Zhang F, Hu H, et al. Integration of summary data from GWAS and eQTL studies predicts complex trait gene targets. *Nat Genet* 2016; **48**(5): 481-7.

23. Gusev A, Ko A, Shi H, et al. Integrative approaches for large-scale transcriptome-wide association studies. *Nature Genetics* 2016; **48**(3): 245-52.

24. Zhang W, Voloudakis G, Rajagopal VM, et al. Integrative transcriptome imputation reveals tissue-specific and shared biological mechanisms mediating susceptibility to complex traits. *Nature Communications* 2019; **10**(1): 3834.

25. Freytag V, Vukojevic V, Wagner-Thelen H, et al. Genetic estimators of DNA methylation provide insights into the molecular basis of polygenic traits. *Translational Psychiatry* 2018; **8**(1): 31.

26. Bhattacharya A, Li Y, Love MI. MOSTWAS: Multi-Omic Strategies for Transcriptome-Wide Association Studies. *PLoS Genet* 2021; **17**(3): e1009398.

27. Barbeira AN, Dickinson SP, Bonazzola R, et al. Exploring the phenotypic consequences of tissue specific gene expression variation inferred from GWAS summary statistics. *Nature Communications* 2018; **9**(1): 1825.

28. Westerman KE, Pham DT, Hong L, et al. GEM: scalable and flexible gene–environment interaction analysis in millions of samples. *Bioinformatics* 2021; **37**(20): 3514-20.

29. Lin D-Y, Tao R, Kalsbeek William D, et al. Genetic Association Analysis under Complex Survey Sampling: The Hispanic Community Health Study/Study of Latinos. *The American Journal of Human Genetics* 2014; **95**(6): 675-88.

30. Lin WY. Detecting gene-environment interactions from multiple continuous traits. *Bioinformatics* 2024; **40**(7).

31. Luo L, Mehrotra DV, Shen J, Tang ZZ. Multi-trait analysis of gene-by-environment interactions in large-scale genetic studies. *Biostatistics* 2024; **25**(2): 504-20.

32. Majumdar A, Burch KS, Haldar T, et al. A two-step approach to testing overall effect of gene-environment interaction for multiple phenotypes. *Bioinformatics* 2021; **36**(24): 5640-8.

33. Kerin M, Marchini J. Inferring Gene-by-Environment Interactions with a Bayesian Whole-Genome Regression Model. *The American Journal of Human Genetics* 2020; **107**(4): 698-713.

34. Rohart F, Gautier B, Singh A, KA LC. mixOmics: An R package for 'omics feature selection and multiple data integration. *PLoS Comput Biol* 2017; **13**(11): e1005752.

35. Argelaguet R, Velten B, Arnol D, et al. Multi-Omics Factor Analysis-a framework for unsupervised integration of multi-omics data sets. *Mol Syst Biol* 2018; **14**(6): e8124.

36. Meng C, Kuster B, Culhane AC, Gholami AM. A multivariate approach to the integration of multi-omics datasets. *BMC Bioinformatics* 2014; **15**: 162.

37. Wang T, Shao W, Huang Z, et al. MOGONET integrates multi-omics data using graph convolutional networks allowing patient classification and biomarker identification. *Nat Commun* 2021; **12**(1): 3445.

38. Brière G, Darbo É, Thébault P, Uricaru R. Consensus clustering applied to multi-omics disease subtyping. *BMC Bioinformatics* 2021; **22**(1): 361.

39. Poirion OB, Jing Z, Chaudhary K, Huang S, Garmire LX. DeepProg: an ensemble of deep-learning and machine-learning models for prognosis prediction using multi-omics data. *Genome Medicine* 2021; **13**(1): 112.

40. Wen Y, Song X, Yan B, et al. Multi-dimensional data integration algorithm based on random walk with restart. *BMC Bioinformatics* 2021; **22**(1): 97.

41. Dimitrakopoulos C, Hindupur SK, Häfliger L, et al. Network-based integration of multi-omics data for prioritizing cancer genes. *Bioinformatics* 2018; **34**(14): 2441-8.

42. Wang SW, Herriges MJ, Hurley K, Kotton DN, Klein AM. CoSpar identifies early cell fate biases from single-cell transcriptomic and lineage information. *Nat Biotechnol* 2022; **40**(7): 1066-74.

43. Wu S, Xu Y, Zhang Q, Ma S. Gene-environment interaction analysis via deep learning. *Genet Epidemiol* 2023; **47**(3): 261-86.
